# Safety of Simultaneous Coronary Artery Bypass Grafting and Carotid Endarterectomy Versus Isolated Coronary Artery Bypass Grafting: A Randomized Clinical Trial

**DOI:** 10.1161/STROKEAHA.117.017570

**Published:** 2017-09-15

**Authors:** Christian Weimar, Konstantinos Bilbilis, Jan Rekowski, Torulv Holst, Friedhelm Beyersdorf, Martin Breuer, Manfred Dahm, Anno Diegeler, Arne Kowalski, Sven Martens, Friedrich W. Mohr, Jiri Ondrášek, Beate Reiter, Peter Roth, Ralf Seipelt, Markus Siggelkow, Gustav Steinhoff, Anton Moritz, Mathias Wilhelmi, Gerhard Wimmer-Greinecker, Hans-Christoph Diener, Heinz Jakob, Claudia Ose, Andre Scherag, Stephan C. Knipp

**Affiliations:** From the Universitätsklinikum Essen, Klinik für Neurologie, Germany (C.W., H.-C.D.); Universitätsklinikum Essen, Zentrum für Klinische Studien (ZKSE), Germany (K.B., C.O.); Universitätsklinikum Essen, Institut für Medizinische Informatik, Biometrie und Epidemiologie (IMIBE), Germany (J.R.); Universitätsklinikum Essen, Klinik für Thorax- und Kardiovaskuläre Chirurgie, Germany (T.H., H.J., S.C.K.); Universitäts-Herzzentrum Freiburg-Bad Krozingen, Klinik für Herz- und Gefäßchirurgie, Germany (F.B.); Universitätsklinikum Jena, Klinik für Herz- und Thoraxchirurgie, Jena, Germany, now Zentralklinik Bad Berka, Germany (M.B.); Westpfalz-Klinikum, Klinik für Thorax-, Herz- und Gefäßchirurgie, Kaiserslautern, Germany (M.D.); Herz- und Gefäßklinik Bad Neustadt/Saale, Klinik für Kardiochirurgie, Bad Neustadt an der Saale, Germany (A.D.); Universitätsklinikum Schleswig-Holstein, Campus Kiel, Klinik für Herz- und Gefäßchirurgie, Kiel, Germany (A.K., M.S.); Universitätsklinikum Münster, Klinik für Herzchirurgie, Germany (S.M.); Herzzentrum Leipzig, Universitätsklinik, Klinik für Herzchirurgie, Leipzig, Germany (F.W.M.); Centrum Kardiovaskulární a Transplantační Chirurgie, Brno, Czech Republic (J.O.); Universitäres Herzzentrum Hamburg, Klinik und Poliklinik für Herz- und Gefäßchirurgie, Germany (B.R.); Universitätsklinikum Gießen und Marburg, Herz-, Kinderherz- und Gefäßchirurgie, Giessen, Germany (P.R.); Universitätsmedizin Göttingen, Abteilung für Thorax-,Herz und Gefäßchirurgie, Göttingen, Germany, now SHG Kliniken Völklingen, Klinik für Herz- und Thoraxchirurgie, Völklingen, Germany (R.S.); Imland Klinik, Gefäß- und Thoraxchirurgie, Rendsburg, Germany (M.S.); Universität Rostock, Klinik und Poliklinik für Herzchirurgie, Germany (G.S.); Johann Wolfgang-Goethe-Universität, Klinik für Thorax-,Herz- und thorakale Gefäßchirurgie, Frankfurt, Germany (A.M.); Medizinische Hochschule Hannover, Klinik für Herz-, Thorax-, Transplantations- und Gefäßchirurgie, Hannover, Germany (M.W.); Herz- und Gefäßzentrum Bad Bevensen, Klinik für Herz-Thorax-Chirurgie, Bad Bevensen, Germany (G.W.-G.); and Universitätsklinikum Jena, Klinische Epidemiologie; Center for Sepsis Control and Care (CSCC), Germany (A.S.).

**Keywords:** carotid stenosis, coronary artery bypass, endarterectomy, carotid, randomized controlled trial, stroke

## Abstract

**Background and Purpose—:**

The optimal operative strategy in patients with severe carotid artery disease undergoing coronary artery bypass grafting (CABG) is unknown. We sought to investigate the safety and efficacy of synchronous combined carotid endarterectomy and CABG as compared with isolated CABG.

**Methods—:**

Patients with asymptomatic high-grade carotid artery stenosis ≥80% according to ECST (European Carotid Surgery Trial) ultrasound criteria (corresponding to ≥70% NASCET [North American Symptomatic Carotid Endarterectomy Trial]) who required CABG surgery were randomly assigned to synchronous carotid endarterectomy+CABG or isolated CABG. To avoid unbalanced prognostic factor distributions, randomization was stratified by center, age, sex, and modified Rankin Scale. The primary composite end point was the rate of stroke or death at 30 days.

**Results—:**

From 2010 to 2014, a total of 129 patients were enrolled at 17 centers in Germany and the Czech Republic. Because of withdrawal of funding after insufficient recruitment, enrolment was terminated early. At 30 days, the rate of any stroke or death in the intention-to-treat population was 12/65 (18.5%) in patients receiving synchronous carotid endarterectomy+CABG as compared with 6/62 (9.7%) in patients receiving isolated CABG (absolute risk reduction, 8.8%; 95% confidence interval, −3.2% to 20.8%; *P*_WALD_=0.12). Also for all secondary end points at 30 days and 1 year, there was no evidence for a significant treatment-group effect although patients undergoing isolated CABG tended to have better outcomes.

**Conclusions—:**

Although our results cannot rule out a treatment-group effect because of lack of power, a superiority of the synchronous combined carotid endarterectomy+CABG approach seems unlikely. Five-year follow-up of patients is still ongoing.

**Clinical Trial Registration—:**

URL: https://www.controlled-trials.com. Unique identifier: ISRCTN13486906.

## Abstract 2

Supplemental Digital Content is available in the text.

**See related article, p 2650**

Coronary artery bypass graft (CABG) surgery is the most commonly performed major cardiovascular operation. Carotid artery stenosis is present in ≈6% to 8% of all patients undergoing CABG and is associated with an increased risk of stroke during and after CABG.^1,2^ Prophylactic treatment of asymptomatic concomitant carotid artery stenosis is managed in different ways, for example, by carotid artery angioplasty and stenting (CAS) or carotid endarterectomy (CEA), either simultaneously with CABG, before CABG, or delayed after CABG (staged or reverse staged). For many years, staged or synchronous CEA has been advocated by many cardiovascular surgeons in the attempt to reduce the perioperative and long-term risk of stroke associated with carotid artery stenosis but only very few patients with this disease entity have been included in controlled clinical trials.^3,4^ Only data from uncontrolled studies with variable inclusion criteria and end point assessment are available in patients with coexisting cardiac and carotid atherosclerotic disease undergoing CABG without carotid revascularization (by CAS or CEA).^5^ Some studies have even found that asymptomatic carotid stenosis did not increase the risk of post-CABG stroke.^6,7^ Therefore, in the absence of any randomized controlled trial, no systematic high-level evidence exists that staged or synchronous CEA and CABG confer any short-term benefit over CABG without CEA. Moreover, improvements in medical therapy have considerably reduced the average long-term risk of ipsilateral stroke in patients with asymptomatic carotid stenosis.^8^ Thus, any potential long-term benefit conferred by prophylactic CEA may be offset by the relatively high procedural risk reported in systematic reviews.^9,10^ Therefore, the CABACS trial (Coronary Artery Bypass Graft Surgery in Patients With Asymptomatic Carotid Stenosis) aimed to compare the perioperative safety and long-term efficacy of synchronous CEA and CABG versus isolated CABG in patients with asymptomatic high-grade carotid artery stenosis.

## Methods

### Study Design

This investigator-initiated trial was designed as a multicenter, randomized (one-to-one), open, group sequential trial with 2 parallel arms and blinded end point adjudication. After initial commitment of 35 major German cardiac surgery centers, 25 German and 1 Czech center could be initiated and 17 centers finally recruited. Ethics approval was obtained from each center. The trial was conducted according to Good Clinical Practice guidelines and the principles stated in the latest revision of the Declaration of Helsinki. The trial protocol has been described previously.^11^ Two amendments to the protocol were implemented in 2012 and 2014 and approved by the local ethics committees of each participating center (online-only Data Supplement). Enrollment was terminated early because of withdrawal of funding following insufficient recruitment.

### Participants

The inclusion criteria were as follows: asymptomatic (past 180 days) internal carotid artery stenosis ≥80% (following criteria of the ECST [European Carotid Surgery Trial],^12^ main criterion: in-stenosis peak systolic velocity ≥300 cm/s, corresponding to ≥70% NASCET [North American Symptomatic Carotid Endarterectomy Trial]^13,14^), carotid artery stenosis treatable with CEA, negative pregnancy test in premenopausal women, written informed consent and full legal capacity, ability of the patient to participate in follow-up examinations.

Exclusion criteria were as follows: nonatherosclerotic stenosis (eg, dissection, floating thrombus, fibromuscular dysplasia, tumor, and postradiation), complete occlusion or previous stenting of the carotid artery to be treated, additional higher grade intracranial or intrathoracic stenosis (tandem stenosis), recent (past 180 days) ischemic symptoms ipsilateral to carotid stenosis or occlusion, contralateral carotid occlusion or other known indication for carotid revascularization (apart from scheduled CABG), myocardial infarction (non–ST-segment–elevation myocardial infarction or ST-segment–elevation myocardial infarction) within the past 7 days (reduced to 48 hours for non–ST-segment–elevation myocardial infarction after the first amendment) or hemodynamically unstable patients, known high risk for cardiogenic embolism requiring anticoagulation (mechanical heart valve, chronic atrial fibrillation [omitted after the first amendment], left ventricular thrombus, left ventricular aneurysm), evidence for intracranial bleeding within the past 90 days, modified Rankin Scale score of >3 or severe aphasia, patients unlikely to survive >1 year because of concomitant diseases, planned combined cardiac valve replacement or any other cardiac surgery beyond CABG (±CEA) during the procedure, major surgery (apart from study procedures) planned within 8 weeks from randomization, and participation in another clinical trial. Written informed consent was obtained from each patient before trial participation.

### Randomization

Eligible patients were randomized to either isolated CABG or synchronous CEA + CABG. To achieve comparable groups, patients were allocated in a concealed way by central preoperative randomization ≥1 day before surgery. To avoid unbalanced prognostic factor distributions, we used a web-based stratified block randomization (strata: center, age [(<60 or ≥60 years], sex [male or female], modified Rankin Scale [score of 0–1 or 2–3]) with randomly varying the block size.

### Interventions

The eligibility of a patient was determined by both a certified study surgeon and a certified study neurologist with experience in cerebrovascular ultrasound examination. Additional imaging of the brain or cerebral circulation was not required or documented as part of the study. CABG with or without CEA under treatment with aspirin was to be performed as soon as possible (maximum within 7 days) after randomization. Standards for surgical treatment were formulated by a surgical quality subcommittee, which also had to approve every surgeon for the study. Each (cardio-) vascular surgeon had to meet the following standards to be certified: anonymous confirmation of the last 30 consecutively performed CEA surgeries, affirmed by the head of department, alone or in combination with anonymous confirmation of the last 150 consecutively performed CABG surgeries, affirmed by the head of department. With regard to carotid revascularization, no policies were prescribed for the use of a shunt, the type of patches, and the use of neurological monitoring or the method of carotid reconstruction (eversion endarterectomy or thromboendarterectomy). With regard to coronary revascularization, the use of extracorporeal circulation (on- or off-pump CABG) was left to the surgeon`s decision. Standards for medical treatment were formulated by a Best Medical treatment subcommittee.

Patients were followed up after 7 days (±1 day), 30 days (±3 days), and 1 year (±30 days) with a neurological examination including evaluation on the National Institutes Health Stroke Scale and the modified Rankin Scale at each visit as well as cerebrovascular ultrasound and DemTect at 30 days and 1 year. The DemTect is a generic dementia screening test, which consists of 5 subtests: a word list, a number transcoding task, a word fluency task, digit span reverse, and delayed recall of the word list. The transformed total score (maximum 18) is corrected for age and does not show any ceiling effect.^15^ A parallel test version was used at 30 days to avoid retest effects.^16^ In addition, a yearly telephone follow-up for 5 years after the operation was also prespecified and is still ongoing.

### Outcomes

The primary composite outcome was the rate of any stroke or death of any cause up to 30 days after the operation (synchronous CEA and CABG versus isolated CABG) or 30 days after randomization for patients not receiving surgery (protocol violations). Stroke was clinically defined as any persistent focal or global neurological deficit lasting longer than 24 hours and presumed to be of no other than vascular origin. All perioperative stroke events were assessed by a study neurologist.

Secondary end points are listed in the Table I in the online-only Data Supplement. For the sake of comparability with previous research, we also included a post hoc secondary composite outcome event of any stroke, myocardial infarction, or death.

All suspected strokes, myocardial infarctions, and all deaths were adjudicated by an independent end point committee, which was blinded to treatment allocation.

### Data Management

Data on paper case report forms were collected at the ZKSE in Essen. Good Clinical Practice compliant remote data capture (Oracle Clinical) was used for entering, managing, and validating the data from all centers.

### Statistical Methods

We initially based the target sample size of 2 groups with 580 participants (total planned sample size 1160) on an estimate of 8.5% frequency of the primary outcome of stroke or death in the synchronous CEA and CABG arm, a clinically relevant 4.5% absolute reduction of this risk to 4.0% in the isolated arm, resulting in 84% power at a 2-sided level of significance of 0.05. The sample size of the first planned interim analysis was 550 (for details see^8^). In accordance with the second amendment, the first interim analysis was performed after the first 100 patients, as recommended by the Data Safety Monitoring Board, and the final analysis was planned after inclusion of 300 patients. A generalized linear mixed-effects model that includes the fixed factors treatment group and the randomization factors age (<60 or ≥60 years), sex (male or female), modified Rankin Scale (score of 0–1 or 2–3), and a random (intercept) factor center was used to test and estimate the effect on the primary end point. This preplanned, confirmatory analysis of the primary end point was based on the Wald test statistic with corresponding 2-sided *P* value for the treatment effect and was applied to the intention-to-treat population. Because of the reduced sample size, we did not apply the originally planned group-sequential design with 1 interim analysis but instead performed 1 final confirmatory analysis at a significance level α of 5%. For robustness, we also ran sensitivity analyses using exact Monte Carlo estimation for χ^2^ tests and analyses excluding major protocol violations (defined in the statistical analysis plan as not meeting any inclusion or exclusion criteria or not finishing the allocated therapy [including change of treatment group, an operation done by a noncertified surgeon, an end point event between randomization and surgical treatment]). For all other exploratory analyses, we also applied an exploratory significance level α of 5% for χ^2^, log-rank, and Wilcoxon–Mann–Whitney tests but focused more on point (mean, relative risk, and hazard ratio) and interval estimators of effects (95% confidence intervals). Details on the biometric analyses—especially those pertaining to the secondary end points—were defined in the statistical analysis plan before data bank closure. Analyses were performed using SAS version 9.4.

### Role of Funding Source

The funder of the study had no role in study design, data collection, data analysis, data interpretation, or writing of the report. The corresponding author had full access to all data from the trial and had final responsibility for the results on submission for publication.

## Results

**Figure 1.**
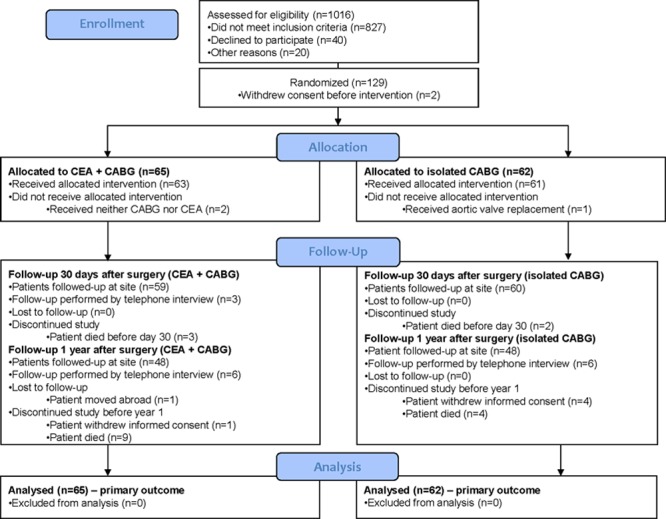
CONSORT flow diagram (Consolidated Standards of Reporting Trials) of patients in the CABACS (Coronary Artery Bypass Graft Surgery in Patients With Asymptomatic Carotid Stenosis) trial. CABG indicates coronary artery bypass grafting; and CEA, carotid endarterectomy.

Between December 2010 and December 2014, 129 patients were randomized in 17 centers with an average recruitment rate of 2.6 per year. Two patients withdrew consent between randomization and treatment and were excluded from the analysis. The intention-to-treat population consisted of 127 patients who were allocated to the 2 arms (Figure 1). Baseline characteristics of both treatment groups are shown in Table 1. Two patients in the synchronous CEA and CABG group did receive neither CABG nor CEA and had no outcome events up to 1 year. One patient in the isolated CABG arm received aortic valve replacement instead of CABG and likewise had no outcome events up to 1 year. Two patients with perioperative ipsilateral ischemic events (1 stroke and 1 TIA) received CAS at 27 and 92 days after isolated CABG. Two patients in the isolated CABG group received CAS of asymptomatic carotid stenosis >30 days after CABG, and 3 patients received CEA of asymptomatic carotid stenosis >30 days after CABG with no subsequent outcome events.

**Table 1.**
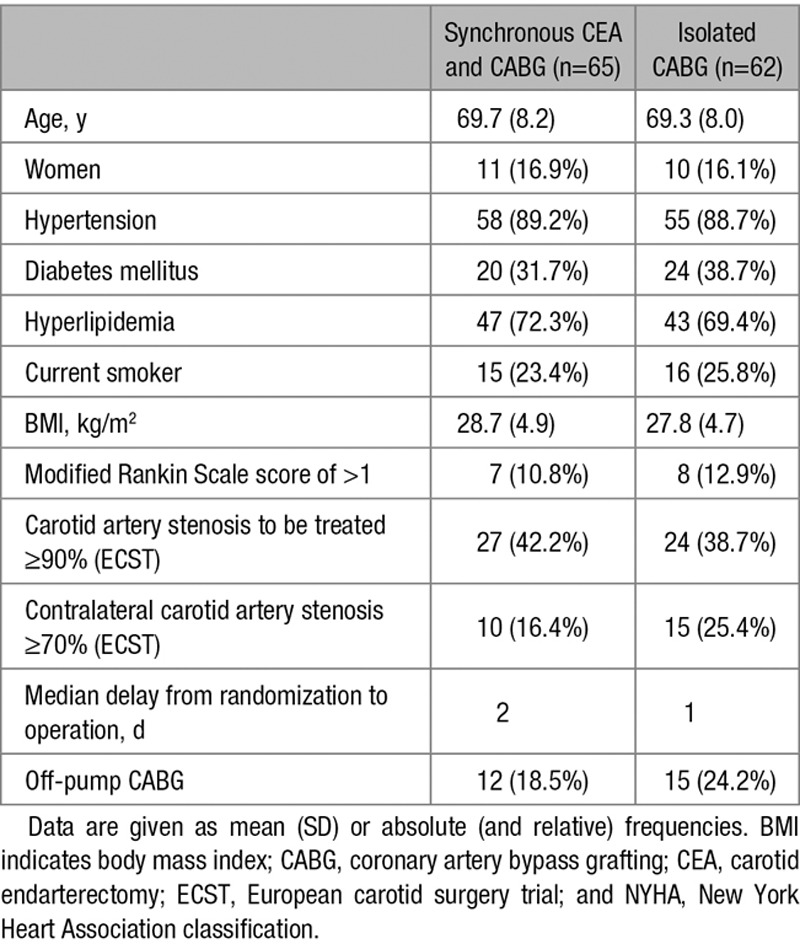
Baseline Characteristics of the Patients Included in the Intention-to-Treat Population

The rates of the primary composite end point of any stroke or death from any cause within 30 days after operation in the intention-to-treat population were 12/65 (18.5%) in the patients who underwent synchronous CEA and CABG and 6/62 (9.7%) in the patients who underwent isolated CABG (absolute risk reduction, 8.8%; 95% confidence interval, −3.2% to 20.8%; *P*_WALD_=0.12). The following major protocol violations resulted in exclusion from the per-protocol analysis: operation by a noncertified surgeon (n=11), myocardial infarction before CABG (n=1), informed consent not provided (n=1), no CABG performed (n=3), prior stenting of carotid stenosis to be treated according to the study protocol (n=1), and no high-grade carotid artery stenosis (n=2). In the per-protocol analysis, primary composite end point event rates at 30 days were 11 of 56 patients (19.6%) who received synchronous CEA and CABG and 6 of 53 patients (11.3%) who received isolated CABG (absolute risk reduction, 8.3%; 95% confidence interval, −5.1% to 21.8%; *P*_WALD_=0.21).

**Figure 2.**
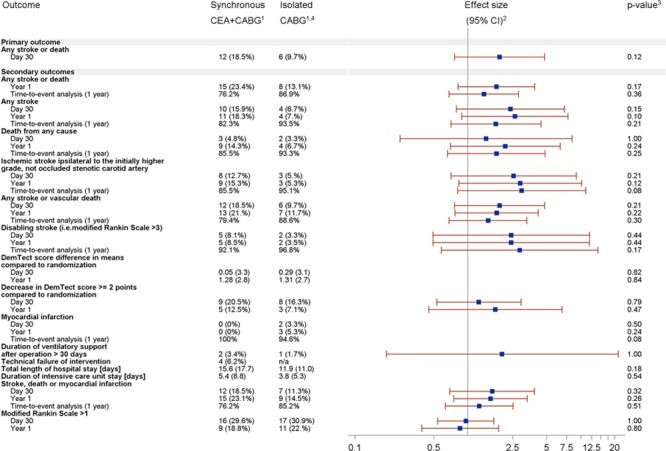
Secondary end points at 30 days and 1 year (forest plot of risk ratios and hazard ratios plotted on a logarithmic scale). ^1^For day 30 and year 1, absolute and relative frequencies; for time-to-event analysis, 1-year Kaplan–Meier estimates; for length of hospital and ICU stay, mean and SD. ^2^For day 30 and year 1, relative risk; for time-to-event analysis, unadjusted hazard ratios for treatment variable from Cox proportional hazards regression; missing effect sizes either not available or not calculated; ^3^Confirmatory analysis of the primary endpoint was based on the Wald test statistic; for day 30 and year 1, exact Monte Carlo estimation for χ^2^ test *P* values; for time-to-event analysis, log-rank test *P* values; for DemTect scale difference, length of hospital stay and ICU stay exact Wilcoxon–Mann–Whitney test *P* values. ^4^Technical failure of intervention can only be measured for the synchronous carotid endarterectomy (CEA) and coronary artery bypass grafting (CABG) arm. CI indicates confidence interval.

Similarly, there was no evidence for a significant treatment-group effect for all secondary end points at 30 days and 1 year (Figure 2) although absolute event rates for isolated CABG were lower for most secondary end points. Sensitivity analyses, showing the same trend but no significant differences, in the per-protocol population are provided in the Table I in the online-only Data Supplement.

**Figure 3.**
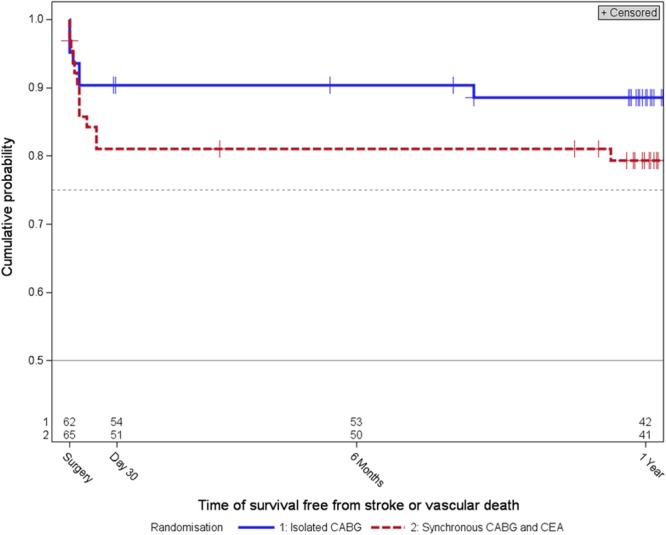
Kaplan–Meier estimates of survival free from stroke or vascular death up to 1 year in the intention-to-treat population (*P*_log-rank_=0.30). CABG indicates coronary artery bypass grafting; and CEA, carotid endarterectomy.

A complete clinical follow-up after 1 year could be obtained in 54 (83.1%) patients in the synchronous CEA and CABG arm and in 56 (90.3%) patients in the isolated CABG arm. Reasons for premature study termination by treatment group are displayed in Figure 1. All patients were on antithrombotic treatment, mostly aspirin, after 1 year and concomitant treatment was balanced among treatment groups (Table 2). Kaplan-Meier estimates of any stroke or vascular death-free survival after 1 year are shown in Figure 3. Pre-specified subgroup analyses as well as two post hoc analyses on the degree of ipsilateral and presence of contralateral carotid artery stenosis were in line with the main analysis (Table II online-only Data Supplement).

**Table 2.**
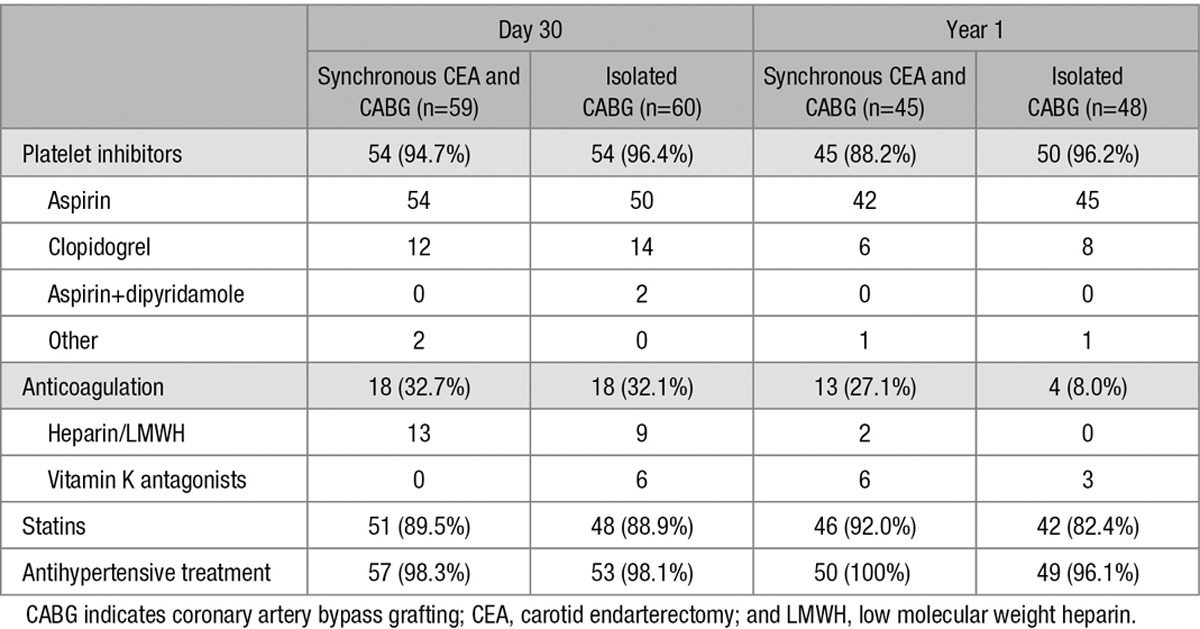
Antithrombotic Treatment During Follow-Up (Patient-Based for Drug Classes, Multiple Responses)

## Discussion

CABACS was conducted as a randomized trial to compare synchronous CEA of asymptomatic high-grade carotid artery stenosis versus no carotid operation in patients undergoing CABG surgery. Because of the low power of the trial following early termination, there was no evidence for a treatment-group effect although patients in the synchronous CEA and CABG arm had double the rate of stroke or death within 30 days or within 1 year compared with CABG without CEA. This observation was also found in predefined subgroups. All secondary end points were also more in favor of the isolated CABG arm but likewise failed to demonstrate a significant difference. Of note, we observed a >2-fold higher overall event rate compared with previously published data, which may result from the relatively high age of our study population, treatment quality, the systematic follow-up by study neurologists, or chance because of the relatively small study sample.^5,17,18^ In contrast, only few patients showed postoperative worsening of cognitive functions as described previously,^19^ but cognitive testing was not possible in all patients, particularly in those with stroke or severe physical impairment.

Our trial is the first multicenter randomized controlled trial with a rigorous design to investigate synchronous CEA versus no carotid operation in patients undergoing CABG. In addition, our trial provides data for perioperative events within 30 days and a 1-year follow-up including cognitive testing. The long-term 5-year follow-up is still ongoing. Two previous (bi- respectively monocentric) RCTs comparing synchronous CEA and CABG with delayed CEA after CABG suggested a lower perioperative risk of stroke in patients undergoing synchronous CEA and CABG compared with delayed CEA after CABG alone.^3,4^ In these studies, however, the very low 30-day risk of stroke or death in the synchronous CEA and CABG arm (1% and 2.8%, respectively) is contradictory to a systematic review and large observational studies and therefore unlikely to represent routine clinical practice.^9,18,20^

Conclusions on the safety of isolated CABG should be made with caution. Although a systematic review and meta-analysis suggested low complication rates in patients with unilateral asymptomatic 70% to 99% carotid artery stenosis undergoing CABG without CEA,^5^ we found a considerably higher rate of perioperative stroke or death in the isolated CABG arm, which may raise concerns also about the isolated CABG approach. However, patient populations (and surgeons) may not have been comparable to other studies and therefore the only way to directly compare different operative strategies versus isolated CABG remains a head-to-head randomized controlled trial. Compared with prior (staged) CEA, a synchronous CEA and CABG approach requires only 1 anesthesia and does not expose patients to the risk of myocardial infarction while waiting for CABG. On the contrary, overall stroke risk seems to be lower with prior staged than with simultaneous CEA and there seems to be no substantial difference in total vascular morbidity and mortality between the 2 approaches.^9,20^ CAS has emerged as a possible alternative to CEA, but most clinical trials in patients with high-grade carotid artery stenosis have shown higher perioperative complication rates for CAS than for CEA, particularly in older men, which constitute the majority of patients undergoing CABG.^21–23^ In a systematic review of cohort studies of predominantly asymptomatic patients with unilateral carotid disease undergoing staged CAS and CABG, the 30-day risk of any stroke or death was 9.1%.^24^ More recent observational studies have even favored CAS before CABG.^20,25,26^ However, dual-antiplatelet therapy is mandated for at least 4 weeks after CAS, which for many surgeons constitutes a reason to postpone CABG because of the risk of bleeding, exposing a patient to an additional risk of myocardial infarction. Whether asymptomatic high-grade carotid artery stenosis unrelated to CABG requires revascularization is the subject of ongoing studies (SPACE-2 [Stent-Protected Angioplasty in Asymptomatic Carotid Artery Stenosis vs Endarterectomy27], ECST-2 [European Carotid Surgery Trial 2; ISRCTN97744893], CREST-2 [Carotid Revascularization and Medical Management for Asymptomatic Carotid Stenosis Trial; NCT02089217])

Our trial has several limitations. The prespecified sample size was not reached because of withdrawal of funding after insufficient recruitment and therefore the study was underpowered to demonstrate statistically significant effects for the minimal, clinically relevant effect expected during the planning phase.^8^ Moreover, investigators were not blinded to treatment allocation. However, main outcome events were adjudicated by blinded observers. Finally, few centers enrolled the large majority of patients, thus limiting the generalizability of our findings. Although all major German cardiovascular centers were invited and the majority participated in this trial, ≈90% of simultaneous CEA and CABG operations in Germany were performed outside of the trial.^17^ A similar problem was encountered in the SPACE-2 trial, which was also stopped early because of slow enrolment.^27^ Although all CABACS centers were required to keep screening logs, only a minority assessed all CABG patients for eligibility and thus bias by selective inclusion remains unknown. Reported reasons for the low study inclusion were surgery preferences of the referring physicians, lack of timely screening for carotid artery stenosis in patients scheduled for CABG, insufficient time to obtain informed consent for study participation, and symptomatic stenosis. Furthermore, an unknown number of patients with asymptomatic carotid artery stenosis scheduled for CABG received staged CEA or CAS before coronary bypass surgery but no registry data or complication rates are available for these patients.

In conclusion, although we cannot rule out a treatment effect, the very high rate of perioperative strokes does not seem to justify simultaneous CEA in patients with high-grade asymptomatic carotid artery stenosis undergoing CABG. Whether any carotid revascularization (CAS or CEA) is warranted in patients with unilateral asymptomatic high-grade carotid artery stenosis requiring CABG remains to be proven. Follow-up studies of the CABACS trial should test staged CAS or CEA followed by CABG versus isolated CABG and be performed in countries with a younger patient population and with reimbursement of staged or synchronous carotid revascularization only if a patient is treated within the trial.

## Acknowledgments

We thank the members of the Data Safety and Monitoring Board for their esteemed support: Peter Ringleb (Universitätsklinikum Heidelberg, Neurologische Klinik, Sektion Vaskuläre Neurologie, Heidelberg, Germany), Hans-Henning Eckstein (Technische Universität München, Klinik und Poliklinik für Vaskuläre und Endovaskuläre Chirurgie, München, Germany), Ross Naylor (University of Leicester, Department of Cardiovascular Sciences, Leicester, United Kingdom), Siegfried Hagl (Universitätsklinikum Heidelberg, Abteilung Herzchirurgie [Emeritus], Heidelberg, Germany), and Andreas Ziegler (Universität zu Lübeck und Universitätsklinikum Schleswig-Holstein, Institut für Biometrie und Statistik, Lübeck, Germany).

## Sources of Funding

The trial was financially supported by the German Research Council (DFG, Bonn, Germany, WE2585-3).

## Disclosures

None.

## Supplementary Material
