# Supplementary material for: Safety of Simultaneous Coronary Artery Bypass Grafting and Carotid Endarterectomy Versus Isolated Coronary Artery Bypass Grafting: A Randomized Clinical Trial
Source: Stroke. 2017 Sep 15;48(10):2769–75. doi: 10.1161/STROKEAHA.117.017570 (PMC5610560; doi:10.1161/STROKEAHA.117.017570)
Supplement: Supplementary file 1 [file str-48-2769-s001.pdf]

SUPPLEMENTAL MATERIAL

**Supplementary table I:** Analyses of secondary outcomes in the per-protocol population*

| Secondary outcome | Time | Synchronous CEA and CABG[1] | Isolated CABG[1] | Effect size (95% CI)[2] | p-value[3] |
|---|---|---|---|---|---|
| Any stroke or death | Day 30 | 11 (19.6%) | 6 (11.3%) | 1.74 (0.69-4.36) | 0.30 |
| | Year 1 | 13 (23.6%) | 8 (15.4%) | 1.54 (0.69-3.40) | 0.34 |
| | Time-to-event analysis (1 year) | 76.8% | 84.6% | 1.10 (0.53-2.29) | 0.80 |
| Any stroke | Day 30 | 9 (16.7%) | 4 (7.8%) | 2.13 (0.70-6.47) | 0.24 |
| | Year 1 | 9 (17.6%) | 4 (8.3%) | 2.12 (0.70-6.42) | 0.24 |
| | Time-to-event analysis (1 year) | 83.8% | 92.4% | 1.30 (0.50-3.42) | 0.59 |
| Death from any cause | Day 30 | 3 (5.6%) | 2 (3.8%) | 1.44 (0.25-8.30) | 1.00 |
| | Year 1 | 8 (14.8%) | 4 (7.8%) | 1.89 (0.61-5.89) | 0.36 |
| | Time-to-event analysis (1 year) | 85.5% | 92.1% | 1.54 (0.56-4.23) | 0.40 |
| Ischemic stroke ipsi-lateral to the initially higher grade, not occluded stenotic carotid artery | Day 30 | 7 (13.0%) | 3 (5.9%) | 2.20 (0.60-8.06) | 0.32 |
| | Year 1 | 7 (14.0%) | 3 (6.3%) | 2.24 (0.61-8.16) | 0.32 |
| | Time-to-event analysis (1 year) | 87.4% | 94.3% | 2.24 (0.58-8.67) | 0.23 |
| Any stroke or vascular death | Day 30 | 11 (19.6%) | 6 (11.3%) | 1.74 (0.69-4.36) | 0.29 |
| | Year 1 | 11 (20.8%) | 7 (13.7%) | 1.51 (0.64-3.60) | 0.44 |
| | Time-to-event analysis (1 year) | 80.4% | 86.6% | 1.16 (0.51-2.65) | 0.72 |
| Disabling stroke (i.e. modified Rankin Scale >3) | Day 30 | 4 (7.5%) | 2 (3.9%) | 1.92 (0.37-10.1) | 0.68 |
| | Year 1 | 4 (8.0%) | 2 (4.2%) | 1.92 (0.37-10.0) | 0.68 |
| | Time-to-event analysis (1 year) | 92.8% | 96.2% | 2.31 (0.45-11.9) | 0.30 |
| DemTect score difference in means compared to randomization | Day 30 | -0.23 (2.9) | 0.55 (3.2) | n/a[4] | 0.36 |
| | Year 1 | 1.18 (2.5) | 1.73 (2.6) | n/a[4] | 0.41 |
| Decrease in DemTect ≥2 points compared to randomization | Day 30 | 8 (20.5%) | 6 (14.3%) | 1.44 (0.55-3.77) | 0.56 |
| | Year 1 | 4 (11.8%) | 2 (5.4%) | 2.18 (0.43-11.1) | 0.42 |
| Myocardial infarction | Day 30 | 0 | 2 (3.9%) | n/a[4] | 0.50 |
| | Year 1 | 0 | 3 (6.3%) | n/a[4] | 0.24 |
| | Time-to-event analysis (1 year) | 100% | 93.6% | n/a[4] | 0.07 |
| Duration of ventilatory support after operation >30 days | | 1 (2.0%) | 1 (2.0%) | 0.98 (0.06-15.2) | 1.00 |
| Technical failure of intervention[5] | | 4 (7.1%) | n/a[4] | n/a[4] | n/a[4] |
| Total length of hospital stay [days] | | 15.7 (18.5) | 12.7 (11.8) | n/a[4] | 0.53 |
| Duration of intensive | | 5.4 (8.3) | 4.2 (5.6) | n/a[4] | 0.77 |

| | | | | | |
|---|---|---|---|---|---|
| care unit stay [days] | | | | | |
| Stroke, death or myocardial infarction | Day 30 | 11 (19.6%) | 7 (13.2%) | 1.49 (0.62-3.55) | 0.44 |
| | Year 1 | 13 (23.2%) | 9 (17.0%) | 1.37 (0.64-2.93) | 0.48 |
| | Time-to-event analysis (1 year) | 76.8% | 82.6% | 0.99 (0.48-2.04) | 0.98 |
| Modified Rankin Scale >1 | Day 30 | 15 (31.3%) | 16 (34.0%) | 0.92 (0.51-1.64) | 0.83 |
| | Year 1 | 7 (17.1%) | 11 (25.6%) | 0.67 (0.29-1.55) | 0.43 |

[1] for day 30 and year 1 absolute and relative frequencies, for time-to-event analysis 1-year Kaplan-Meier estimates, for length of hospital stay and ICU stay mean and standard deviation

[2] for day 30 and year 1 relative risks, for time-to-event analysis unadjusted hazard ratios for treatment variable from Cox proportional hazards regression

[3] for day 30 and year 1 exact Monte Carlo estimation for $\chi^2$ test p-values, for time-to-event analysis log-rank test p-values, for DemTect scale difference, length of hospital stay and ICU stay exact Wilcoxon-Mann-Whitney test p-values

[4] either not available or not calculated

[5] can only be measured for the synchronous CEA and CABG arm

**Supplementary table II:** Rates of the primary composite endpoint for subgroups of predefined clinical variables in the intention-to-treat population (grade of ipsilateral and contralateral carotid artery stenosis was identified *post hoc*).

| | Synchronous CEA and CABG | Isolated CABG | Risk ratio (95% CI) | p-value[1] |
|---|---|---|---|---|
| Sex | | | | |
| Men (n= 106) | 10 (18.5%) | 5 (9.6%) | 1.93 (0.71-5.25) | 0.27 |
| Women (n= 21) | 2 (18.2%) | 1 (10.0%) | 1.82 (0.19-17.1) | 1.00 |
| Age | | | | |
| <60 years (n= 21) | 1 (8.3%) | 0 (0%) | n/a[2] | 1.00 |
| ≥60 years (n= 106) | 11 (20.8%) | 6 (11.3%) | 1.83 (0.73-4.60) | 0.29 |
| Modified Rankin Scale | | | | |
| 0-1 (n= 112) | 10 (17.2%) | 5 (9.3%) | 1.86 (0.68-5.10) | 0.28 |
| 2-3 (n= 15) | 2 (28.6%) | 1 (12.5%) | 2.29 (0.26-20.1) | 0.57 |
| Degree of carotid artery stenosis (ECST) | | | | |
| < 90 % (n= 75) | 6 (16.2%) | 3 (7.9%) | 2.05 (0.55-7.61) | 0.31 |
| ≥ 90 % (n= 51) | 6 (22.2%) | 3 (12.5%) | 1.78 (0.50-6.34) | 0.48 |
| Contralateral carotid disease (ECST) | | | | |
| < 70% (n= 95) | 11 (21.6%) | 4 (9.1%) | 2.37 (0.81-6.92) | 0.16 |
| ≥ 70% (n= 25) | 1 (10.0%) | 2 (13.3%) | 0.75 (0.08-7.21) | 1.00 |

[1] exact Monte Carlo estimation for $\chi^2$ test
[2] either not available or not calculated

CABACS trial investigators
Participating centers (number of patients):
Universitätsklinikum Essen (33): Christian Weimar, Stephan Knipp (PI), Vesna Zegarac, Oliver Kastrup, Heinz Jakob, Johannes Hoffmann, Hans Torulv Holst, Jaroslav Benedik, Anna Cyrek
Universitätsklinikum Hamburg (17): Beate Reiter (PI), Michael Rosenkranz, Christoph Beck, Sebastian Debus, Axel Larena-Avellaneda, Mohammed Ismail
Universitätsklinikum Kiel (13): Markus Siggelkow (PI), Arne Kowalski, Robert Stingele, Jochen Cremer, Justus Groß
Universitäts-Herzzentrum Freiburg-Bad Krozingen (10):Karl-Friedhelm Beyersdorf (PI), Andreas Harloff, Matthias Reinhard, Thomas Winker, Matthias D'Inka, Christoph Haller, Wolfgang Peck, Michael Weinbeck, Joachim Schöllhorn
Universitäres Herzzentrum Jena (9):Martin Breuer (PI), Albrecht Günther, Jens Weise, Carsten Klingner, Tim Sandhaus, Gloria Färber, Mahmoud Diab
Center of cardiovascular surgery and transplantations Brno, CZ (9):Jiri Ondrasek (PI), Michal Reif, Ales Tomasek
Medizinische Hochschule Hannover (8): Mathias Wilhelmi (PI), Karin Weißenborn, Hans Worthmann, Omke E. Teebken, Ingo Kutschka; Malakh Shrestha, Serghei Cebotari; Issam Ismail, Gregor Warnecke, Thomas Aper, Samir Sarikouch
Universitätsklinikum Gießen (8):Peter Roth (PI), Christian Tanislav, Tibo Gerriets, Max Nedelmann, Orhan Coskun, Simon Classen, Ahmed Koshty
Herz- und Gefäßzentrum Bad Bevensen (6):Gerhard Wimmer-Greinecker (PI), Joachim Gerber, Ulrike Heesemann, Andreas Fleischmann, Matthias Machner, Samer Kseibi, Cemal Dogru, Julia Neufeld, Heinz Deschka
Universitätsklinikum Frankfurt (5):Sven Martens, Ulrich Stock (PI), Oliver Singer, Matthias Lorenz, Anton Moritz, Anja Ploss, Arndt-H. Kiessling
Herzzentrum Leipzig (3):Friedrich-Wilhelm Mohr (PI), Joseph Claßen, Johannes Schwarz, Carsten Hobohm, Andreas Schulz, Wolfgang Kloppig, Martin Misfeld, Klaus Bomke, Anne-Kathrin Funkat
Universitätsklinikum Rostock (3):Gustav Steinhoff (PI), Uwe Walter, Stephan Kolbaske, Bernd Westphal, Carsten Bünger, Peter Donndorf, Christian Klopsch, Alexander Kaminski
Universitätsklinikum Göttingen (2):Ralf Seipelt (PI), George Trendelenburg, Paul Lingor, Tomislav Stojanovic, Dieter Zenker
Universitätsklinikum Münster (1): Sven Martens (PI), Erich-B. Ringelstein, Ralf Dittrich, Giovanni Torsello, Dagmar Bachhuber, Thomas Schönefeld, Birgit Wulff, Bernd Kasprzak, Julia Hillebrand
Westpfalz-Klinikum Kaiserslautern (1):Manfred Dahm (PI), Martin Morgenthaler, Yogesh Shah, Erich Haußmann, Florian Sutter
Herz- und Gefäßklinik Bad Neustadt/Saale (1): Anno Diegeler (PI), Bernd Griewing, Hassan Soda, Volker Ziegler, Paul Urbanski, Bert Rosada, Miroslav Pytlik, Rüdiger Guth, Renate Weinhardt

Members of the Steering Committee and Executive Steering Committee (marked with ‘*’):
C. Weimar *(Neurology, University of Duisburg-Essen, Principal Investigator, LKP)
S. C. Knipp * (Thoracic and Cardiovascular Surgery, University of Duisburg-Essen, Coordinating Investigator)
A. Scherag * (Center for Clinical Trials, University of Duisburg-Essen, trial statistician)
F. Beyersdorf (Cardiac and Vascular Surgery, University of Freiburg)
J. Cremer (Cardiac and Vascular Surgery, University of Kiel)
H.-C. Diener (Neurology, University of Duisburg-Essen)
A. Haverich (Heart, Thoracic, Transplantation and Vascular Surgery, Medical School of Hannover)
H. G. Jakob (Thoracic and Cardiovascular Surgery, University of Duisburg-Essen)
F.W. Mohr (Cardiac surgery, University of Leipzig)
C. Ose (Center for Clinical Trials Essen, University of Duisburg-Essen)
H. Reichenspurner (Cardiac and Vascular Surgery, University of Hamburg)
A. Welz (Cardiac surgery, University of Bonn)

# CLINICAL TRIAL PROTOCOL

# CABACS

## Coronary Artery Bypass graft surgery

## in patients with

## Asymptomatic Carotid Stenosis

## A Randomized Controlled Clinical Trial

**Coordinating Investigator (LKP):**
Prof. Dr. med. Christian Weimar
Department of Neurology; University of Duisburg-Essen
Hufelandstr. 55; 45147 Essen
Phone: 0201 723 2495; Fax: 0201 723 5919
e-mail: christian.weimar@uk-essen.de

**TRIAL COORDINATOR**
Dr. med. Stephan Knipp
Dept. of Thoracic and Cardiovascular Surgery; University of Duisburg-Essen
Hufelandstr. 55; 45147 Essen
Phone: 0201 723 4915; Fax: 0201 723 5451
e-mail: stephan.knipp@uk-essen.de

| | |
|---|---|
| Clinical Trial Code: | CABACS |
| DRKS Nummer: | DRKS00000521 |
| Clinical Phase: | Therapeutic confirmatory (Phase III) |
| Version: | 3.1, dated 29.9.2010 |

# CLINICAL TRIAL PROTOCOL SUMMARY

Title: CABACS - Coronary Artery Bypass graft surgery in patients with Asymptomatic Carotid Stenosis. A Randomized Clinical Trial

Phase: Therapeutic confirmatory (Phase III)

**Sponsor:**
University of Duisburg-Essen, Campus Essen - faculty of medicine-, represented by the Board of Directors, which is in turn represented by the Director of Administration Reinhold Keil
Hufelandstr. 55, 45147 Essen
Phone: 0201 / 723-2600
Fax: 0201 / 723-5913
e-mail: reinhold.keil@uk-essen.de

**Principal Investigators:**

**Coordinating Investigator (LKP) and designee of the sponsor**
Prof. Dr. med. Christian Weimar
Department of Neurology; University of Duisburg-Essen
Hufelandstr. 55; 45147 Essen
Phone: 0201 723 2495; Fax: 0201 723 5919
e-mail: christian.weimar@uk-essen.de

**Trial coordinator**
Dr. med. Stephan Knipp
Dept. of Thoracic and Cardiovascular Surgery; University of Duisburg-Essen
Hufelandstr. 55; 45147 Essen
Phone: 0201 723 4915; Fax: 0201 723 5451
e-mail: stephan.knipp@uk-essen.de

**Trial statistician**
Dr. rer. physiol. André Scherag
Center for Clinical Trials Essen (ZKSE), University of Duisburg-Essen
Hufelandstr. 55, 45147 Essen
Phone: 0201 723 4793; Fax: 0201 723 5933
e-mail: andre.scherag@uk-essen.de

**Financing / Status of the Sponsor:**
DFG (WE 2858/3-1) / Non commercial clinical trial

**Indication**:
Coronary artery disease combined with asymptomatic high-grade carotid artery stenosis (ICD I25.X, I65.2)

**Key Inclusion and Exclusion criteria:**
**Key inclusion criteria:**

- Identification of a ≥80% stenosis (ECST criteria) of the extracranial carotid artery in a patient scheduled for coronary artery bypass graft (CABG) surgery
- Written informed consent

**Key exclusion criteria:**

- Stenosis related neurological symptoms within the previous 180 days
- Non-atherosclerotic origin of stenosis
- Myocardial infarction (NSTEMI or STEMI) within 7 days
- Modified Rankin Scale score >3 or severe aphasia

**Objective**:
To compare the safety and efficacy of isolated CABG with synchronous CABG and carotid endarterectomy (CEA) in patients with asymptomatic high-grade carotid artery stenosis

**Trial Design**:
Randomized, controlled, open, multi-center group sequential trial with 2 parallel groups and blinded observers

**Sample Size:**
To be allocated to trial (n = 1,160)
To be analyzed (ITT) (n = 1,100)

**Primary endpoint:**

The primary efficacy endpoint is the event rate of nonfatal strokes or deaths from any cause (whatever occurs first) within 30 days after the intervention (either isolated CABG or synchronous CABG + CEA).

**Secondary endpoints:**

Secondary efficacy endpoints are single components of the primary endpoint, myocardial infarction, technical failures, duration of ventilatory support, change of cognitive performance on the Demtec scale and observations at different time points.

**Statistical Analysis:**

**Efficacy:** The null hypothesis of the primary endpoint is that the event rates for isolated CABG and synchronous CABG + CEA procedure are equal. This hypothesis will be tested with a generalized linear mixed effects model including covariates. The null hypothesis can be rejected if the p-value related to the Wald test statistic for the treatment effect is smaller than either $\alpha_1 = 0.0052$ at the first planned interim analysis (550 patients randomized) or smaller than $\alpha_2 = 0.0480$ at the final analysis of all data (O'Brien and Fleming group sequential plan). The total $\alpha$ is 0.05 (two-sided). The primary analysis will be performed on the intention-to-treat analysis set, which includes all randomized patients. In addition, we will perform analyses on the per-protocol set to check the consistency of the claim.
**Safety:** Safety analyses will be done for all adverse and serious adverse events reported and documented during the trial period. Details will be provided in the

Statistical Analysis Plan (SAP).

**Secondary endpoints:** All secondary analyses will be done exploratively, i.e., without adjustment for multiplicity. Details on the statistical techniques will be provided in the SAP.

**Trial Duration and Dates:**

**Follow-up per patient:** 1 year with an additional telephone follow-up over 4 years
**First patient in to last patient out:** 8 years (3-year recruiting phase, 1-year follow-up; 4 years telephone follow-up)
**Total trial duration:** 8 years

**Duration of treatment per patient:** CABG with or without CEA is to be performed as soon as possible following randomization (see below). Best medical treatment continues throughout the follow-up period.

## ABBREVIATIONS

| | |
|---|---|
| AE | Adverse Event |
| AMG | Arzneimittelgesetz (German Drug Law) |
| BQS | Bundesgeschäftsstelle Qualitätssicherung gGmbH |
| BDSG | Bundesdatenschutzgesetz |
| BMT | Best medical treatment |
| CABG | Coronary artery bypass grafting |
| CAC | Clinical Adjudication Committee |
| CAS | Carotid artery stenosis |
| CEA | Carotid endarterectomy |
| CI | Confidence interval |
| CRF | Case Report Form |
| CTCAE | Common Toxicity Criteria for Adverse Events |
| CV | Curriculum Vitae |
| DFG | Deutsche Forschungsgemeinschaft (German Research Council) |
| DRKS | Deutsches Register Klinischer Studien (German clinical trials register) |
| DSMB | Data Safety Monitoring Board |
| EC | Ethics Committee |
| ECG | Electrocardiogram |
| GCP | Good Clinical Practice |
| ICH | International Conference on Harmonization of Technical Requirements for Registration of Pharmaceuticals for Human Use |
| ICU | Intensive Care Unit |
| ISF | Investigator Site File |
| ISRCTN | International Standard Randomized Controlled Trial Number |
| ITT | Intention To Treat |
| LKP | Leiter der Klinischen Prüfung (Coordinating Investigator according to AMG) |
| MI | Myocardial infarction |
| n.a. | not applicable |
| OE | Outcome Event |
| SAE | Serious Adverse Event |
| SC | Steering Committee |
| SDV | Source Data Verification |
| TMF | Trial Master File |
| ZKSE | Koordinierungszentrum für Klinische Studien, Essen (Coordination Center for Clinical Trials, Essen) |

# TABLE OF CONTENTS

Clinical Trial Protocol Summary 6
Abbreviations 9
Table of Contents 10
1 Introduction 14
1.1 Scientific background 14
*1.1.1 Evidence 14*
*1.1.2 Role of isolated CABG in patients with asymptomatic CAS 16*
*1.1.3 Role of combined CABG and carotid endarterectomy 16*
*1.1.4 Role of medical management 16*
1.2 Trial rationale / justification 16
1.3 Benefit / risk assessment 17
2 Trial Objectives and Endpoints 17
2.1 General aim / primary objective 17
2.2 Composite primary efficacy endpoint 18
2.3 Assessment of safety 18
2.4 Secondary outcomes 18
3 Trial Design 18
4 Selection of Subjects 19
4.1 Number of subjects 19
4.2 General criteria for subjects' selection 19
4.3 Inclusion criteria 19
4.4 Exclusion criteria 19
4.5 Criteria for withdrawal 20
*4.5.1 Withdrawal of subjects 20*
*4.5.2 Replacement of Subjects 20*
*4.5.3 Premature Closure of the Clinical Trial 21*
5 Trial Procedures 21
5.1 Description of trial days 21
5.2 Screening and randomization 21
*5.2.1 Informed consent 22*
*5.2.2 Risk factor screening 22*
*5.2.3 Neurological examination 22*
*5.2.4 Prior and concomitant illnesses 22*

*5.2.5 Prior and concomitant treatments* *22*
*5.2.6 Ultrasound examination* *22*
5.3 Treatment 23
*5.3.1 Allocation to treatment / Randomization* *23*
*5.3.2 Best medical treatment* *23*
*5.3.3 Isolated CABG without carotid endarterectomy* *23*
*5.3.4 Combined CABG and carotid endarterectomy* *23*
5.4 Blinding 23
5.5 Follow-up examinations 24
5.6 Plan for medical treatment and care after the trial 24
6 Assessments 24
6.1 Definitions 24
6.2 Evaluation of safety 26
6.3 Evaluation of efficacy 26
*6.3.1 Primary endpoint* *26*
*6.3.2 Secondary endpoints* *26*
*6.3.3 Additional analysis* *26*
6.4 Endpoint evaluation 26
7 Complications and Serious Adverse Event reporting 27
7.1 Definitions 27
*7.1.1 Complications* *27*
*7.1.2 Serious adverse event* *28*
7.2 Period of observation and documentation 28
7.3 Reporting of SAEs / OE by investigator 28
7.4 Expedited reporting 29
8 Data Management 29
8.1 Data collection 29
8.2 Data handling 29
8.3 Storage and archiving of data 30
9 Data analysis / statistical methods 30
9.1 Sample size calculation 30
9.2 Definition of trial population to be analyzed 31
9.3 Statistical methods 31
9.4 Interim analyses 32
10 STUDY ADMINISTRATION 32

10.1 Data Safety and Monitoring Board (DSMB) 32
10.2 Steering Committee (SC) and Executive Committee (ExC) 33
10.3 Clinical Adjudication committee (CAC) 33
10.4 Quality Subcommittees 33
10.5 Control of treatment quality 33
*10.5.1 Centers 33*
*10.5.2 Investigators 33*
10.6 Investigator Site File 34

11 ETHICAL AND LEGAL ASPECTS 34
11.1 Good Clinical Practice 34
11.2 Patient information and informed consent 34
11.3 Patient insurance 34
11.4 Confidentiality 35
11.5 Responsibilities of investigator 35
11.6 Approval of trial protocol and amendments 35
11.7 Continuous information to independent ethics committee 36

12 Quality Assurance 36
12.1 Monitoring 36
12.2 Inspections / Audits 37

13 Agreements 37
13.1 Financing of the trial 37
13.2 Publication 37

14 Signatures 38
15 Declaration of Investigator 39
16 References 40

## Flow Chart / Trial Schedule

All patients scheduled for elective CABG surgery are routinely screened for carotid artery stenosis (CAS) in the participating centers. Neurological assessment before surgical intervention will further define study eligibility. Patients willing to participate as documented by written informed consent will be assigned to either treatment group by 1:1 block stratified randomization with variable block length and stratification factors (age, sex, and modified Rankin scale) for each center. Surgical intervention according to group allocation will be performed as soon as possible (but within less than 7 days) by a study-certified surgeon according to best practice. Adverse events occurring during or after surgery are continuously documented during the acute hospital stay.

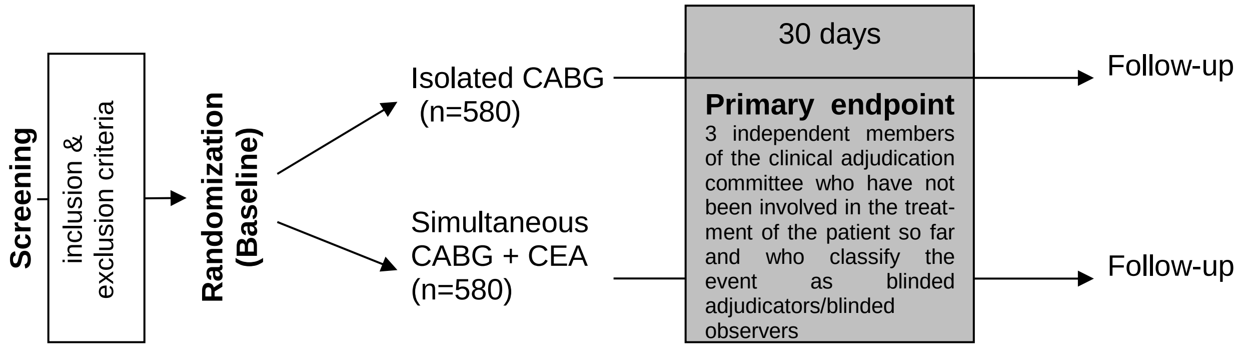


**Figure 1:** Trial flow chart and visit plan

Patients will be followed-up by the study neurologist 7, 30 and 365 days after the procedure. In case of a suspected cerebrovascular event, cerebral imaging is considered clinical standard and therefore mandatory.

| Test | Screening Baseline | CABG / CEA | 7 days (+/-1) | 30 days (+/- 3) | 365 days (+/- 14) | Year 2-5 (+/- 30 d) |
|---|---|---|---|---|---|---|
| Informed Consent | √ | | | | | |
| Medical History | √ | | | | | |
| ECG | √ | | √ | √ | | |
| NIH - Stroke Scale | √ | | √ | √ | √ | |
| Demtec | √ | | | √ | √ | |
| Modified Rankin Scale | √ | | | √ | √ | √ |
| Doppler-/Duplexsonography | √ | | | √ | √ | |
| Complications, Serious adverse events | | √ | √ | √ | | |
| Outcome Events | | √ | √ | √ | √ | √ |
| Prior and concomitant relevant treatments | √ | √ | √ | √ | √ | √ |
| Surgical documentation including BQS | √ | √ | √ | √ | | |

# 1 INTRODUCTION

## 1.1 Scientific background

Annually, more than 60,000 coronary artery bypass graft (CABG) procedures are performed in Germany.[1] In patients without carotid artery stenosis (CAS) undergoing CABG, the perioperative risk of stroke or death is estimated at about 2-3%.[2, 3] Significant CAS is present in approximately 6-8% of all patients undergoing CABG and is associated with an increased risk of stroke during and after CABG.[4, 5] Treatment in these patients (in Germany estimated at 4,000-5,000 per year) is handled controversially. Staged or synchronous carotid endarterectomy is advocated by many cardiothoracic surgeons to reduce the high perioperative and long-term stroke risk associated with polyvascular disease. However, no randomized trial has assessed whether a combined or staged procedure confers any overall benefit when compared with isolated CABG. While the perioperative risk of combined CABG and CEA remains high, the perioperative risk of isolated CABG estimated from single-center case series seems to be considerably lower.[6, 7] Nevertheless, for a systematic review, only insufficient data from uncontrolled studies with variable inclusion criteria and endpoint assessment are available in patients with combined cardiac and carotid disease not undergoing staged or synchronous surgery.[6]

The other point to consider in patients with asymptomatic CAS is the long-term risk of stroke of about 1-2% per year.[8] The long-term prognosis after CEA (without CABG) versus best medical treatment was investigated in the Asymptomatic Carotid Surgery Trial (ACST) and the smaller Asymptomatic Carotid Atherosclerosis Study (ACAS).[9, 10] In a meta-analysis including 5,223 patients with asymptomatic CAS, patients undergoing CEA fared better than those treated medically for the outcome of perioperative stroke or death or any subsequent stroke over 5 years (relative risk 0.69; 95% CI 0.57 to 0.83).[8] Thus, there is a mild superiority of this intervention compared with standard medical therapy, provided that the CEA can be done with a perioperative morbidity and mortality of less than 3%. Even then, about 20 patients have to be treated to prevent one stroke over a period of 5 years, which also causes doubts about the cost effectiveness of this intervention. In addition, the majority of conservatively treated patients was included more than 10 years ago and was not treated according to current recommendations and guidelines. Therefore, two ongoing trials, “Transatlantic Asymptomatic Carotid Intervention Trial” (TACIT) and “Stent–Protected Angioplasty in asymptomatic Carotid artery stenosis” (SPACE-2), are currently re-evaluating therapy in patients with asymptomatic CAS. These trials will thus determine which perioperative morbidity and mortality rates are compatible with a long-term benefit of CEA in patients with asymptomatic CAS.

### 1.1.1 Evidence

CABG was introduced in 1968 and has become one of the most commonly performed major operations, with approximately 800,000 patients each year undergoing this procedure worldwide. CABG provides definite clinical advantage in patients with coronary artery disease, resulting in the relief of angina and an improved quality of life.

Advances in coronary surgery (e.g. off-pump CABG, smaller incisions, enhanced myocardial preservation, use of arterial conduits, and improved postoperative care) have reduced morbidity, mortality, and rates of graft occlusion.[11] Percutaneous coronary intervention (PCI) was introduced in 1977 and is an alternative definite option for treating patients with coronary artery disease. In the past few years, several randomized clinical trials[12] and observational studies[13] have examined outcomes for these interventions, but most, with few exceptions, were conducted before the availability of stenting. New stent devices and improvements in PCI techniques, however, have revolutionized the latter approach and challenge CABG in the treatment of coronary three-vessel disease and/or left main stem disease. Observational data from very large New York cardiac registries comparing short-term and long-term outcomes among patients with multivessel disease who underwent CABG (n=37,312) or stenting (n=22,102) showed that for patients with two or more diseased coronary arteries, CABG is associated with higher adjusted rates of long-term survival and lower rates for repeat revascularization than stenting.[14] In these days, PCI involving drug-eluting stents is increasingly used to treat complex coronary artery disease. In the Synergy between PCI with Taxus and Cardiac Surgery (Syntax) trial, the optimal revascularization strategy for patients with previously untreated three-vessel or left main coronary artery disease is being assessed.[15] Recently, the two-year results of this largest comparative randomized trial became available. These data demonstrated comparably good results in patients with less complex coronary artery disease between PCI and CABG, while patients with more complex coronary pathology had better results by surgical intervention. Hence, in these patients, CABG is regarded as the primary recommendation of revascularization. No ongoing or completed randomized trial has assessed whether a combined synchronous or staged CEA and CABG procedure confers any benefit compared with isolated CABG.[16] A recent systematic review of perioperative outcomes following synchronous CEA and CABG revealed a complication rate of 8.2% (95% CI 7.1-9.3%) for perioperative stroke or death in 5,386 patients.[6] Only limited data are available on other strategies: In 649 predominantly asymptomatic patients undergoing staged carotid artery stenting and CABG, the 30-day risk of any stroke or death was 9.4% (95% CI 7.0-11.8%),[17] whereas it was 6.1% (95% CI 2.9-9.3%) in 709 similar patients undergoing staged CEA and CABG.[6] However, when including myocardial infarction as an endpoint, the combined outcome rate for staged CEA and CABG rises to 10.2% (95% CI 7.4-13.1%). Another registry study that was not included in this review showed an in-patient death / stroke rate of 8.6% after synchronous CEA and CABG in 26,197 patients treated between 2000 and 2004.[18] Because screening for perioperative stroke is not routinely done by neurologists, the stroke rate is likely to be higher than the reported 3.9%. Similarly, propensity-score-matched studies comparing combined CABG and CEA and CABG without prophylactic CEA have reported conflicting results regarding overall morbidity and in-hospital mortality.[19, 20] Because synchronous CEA and CABG procedures still constitute a frequently used approach in many German centers with a well documented periprocedural risk, it was chosen as comparator for isolated CABG and in the absence of a gold standard was defined as the control group.

Given an estimated annual frequency of about 4,000-5,000 CABG procedures in patients with significant CAS and the commitment of the 40 leading cardiothoracic surgery centers in Germany, recruitment of 10-20 patients annually per center seems feasible to meet the study size of 1,160 patients over 3 years.

### 1.1.2 Role of isolated CABG in patients with asymptomatic CAS

Given a prevalence of about 6-8% CAS in patients undergoing CABG surgery and a frequency of about 2% simultaneous operations in administrative databanks on CABG, a considerable number of CABG operations are performed without CEA in patients with asymptomatic CAS.[4] Nevertheless, data on safety and long-term outcome in these patients are very scarce. While no randomized studies have been performed in patients with asymptomatic CAS, the perioperative risk of CABG without CEA or carotid stenting derived from single-center case series was not increased in comparison with CABG in patients without CAS in retrospective case series.[6, 7, 21] In contrast, the only prospective observational study in patients with (symptomatic and asymptomatic) CAS found a higher stroke rate in patients undergoing isolated CABG compared with combined CABG and CEA but lower mortality and lower rate of myocardial infarction.[22]

### 1.1.3 Role of combined CABG and carotid endarterectomy

During the synchronous operation, CEA is usually performed before CABG. Pooled data from single-center observational studies showed a perioperative rate of stroke or death of 8.2% (95% CI 7.1-9.3%),[6] whereas a nationwide US registry reported a risk of 8.6% for stroke or death after synchronous CABG / CEA in 26,197 patients treated between 2000 and 2004.[18] It remains unclear if this increased risk is due to the high vascular comorbidity or the combined CABG / CEA procedure. In a propensity score analysis, Ricotta et al. could not find an increased risk for the combined operation in the New York State Cardiac Data Base,[20] whereas risk adjustment in the nationwide inpatient sample revealed a 38% increased rate of complications in patients undergoing combined CABG / CEA.[23]

Although the long-term benefit of CEA for asymptomatic CAS has been established, ongoing studies will have to demonstrate which perioperative complication rate is compatible with a long-term benefit of CEA (or carotid stenting) compared to modern medical treatment.

### 1.1.4 Role of medical management

Medical management of patients with asymptomatic carotid stenosis consists of risk factor modification, statin therapy, antiplatelet medication and careful monitoring of progression or hemodynamic relevance by ultrasound. These treatment regimens have been the standard therapy for asymptomatic carotid stenosis in the past and many treatment guidelines still recommend only medical therapy if surgical treatment cannot be provided with perioperative morbidity (stroke, MI) and mortality rates below 3%.[24]

## 1.2 Trial rationale / justification

There is an ongoing debate as to which strategy is optimal with regard to the incidence of complications and long-term outcomes after CABG surgery.[3, 25] In the absence of any randomized controlled trials, no systematic evidence exists that staged or synchronous operations confer any benefit over CABG without CEA. Conversely, it remains questionable whether the observed risk of the synchronous procedure is justified in any asymptomatic patient with high-grade carotid stenosis.[6] Thus, any potential (long-term) benefit conferred by prophylactic carotid intervention (CEA or stenting) may be offset by

the relatively high procedural risks observed in systematic reviews. Evidence for isolated CABG, the least invasive and least expensive strategy, is limited to self-reported, uncontrolled, mostly retrospective case series. Only a randomized controlled trial will finally settle this open question and is expected to provide a base for defining an evidence-based standard. It will also enable development and evaluation of improved surgical techniques or strategies and thus have a wide impact on management of this disease. The fact that 40 cardiothoracic centers in cooperation with neurologists in Germany have committed to perform this study is further proof that a randomized trial is desperately needed.

Every individual in the trial will benefit from treatment by certified experts and the supervision of the medical treatment and risk factor management by neurologists. With an estimated 4,000-5,000 CABG procedures performed in patients with significant carotid artery stenosis in Germany, treatment of this condition is frequent. Estimated proceeds of 3,900 € (G-DRG F06A versus F06B) for an additional synchronous or staged CEA / carotid artery stenting performed in roughly 2,000-2,500 patients thus results in an annual burden of around 8-10 million €. This demonstrates that apart from preventing morbidity / mortality, there is a high economic interest in clarifying the optimal treatment strategy.

### 1.3 Benefit / risk assessment

The benefit of each of the two treatment options is the proposed reduction of perioperative and long-term cerebro- and cardiovascular events. However, each treatment option also carries specific risks. Most feared risks of both isolated CABG and combined CABG/CEA are periprocedural stroke and death. Differences between the risks of isolated CABG and combined CABG/CEA are based on technical details. CEA is usually performed before CABG, which contributes to the complication rate related to the combined procedure but possibly lowers the perioperative stroke risk during CABG surgery. In contrast, isolated CABG may put patients at risk of hemodynamic or embolic stroke distal to the CAS. In addition, these patients carry a continued long-term risk of stroke of approximately 1-2% per year. Thus, isolated CABG is expected to go along with possibly lower 30-day complication rates but possibly higher long-term ipsilateral stroke rates. The reverse is to be expected with the combined procedure. To minimize the periprocedural risks, every individual in the trial will be treated by named experts and a close monitoring for such events will be done. A quality subcommittee defines the rules for the treatment and the quality control criteria (see 10.4).

## 2 TRIAL OBJECTIVES AND ENDPOINTS

### 2.1 General aim / primary objective

The objective of this study is to compare safety and efficacy of isolated CABG with combined synchronous CABG and CEA in individuals with asymptomatic atherosclerotic carotid artery stenosis.

### 2.2 Composite primary efficacy endpoint

The primary efficacy endpoint is the event rate of nonfatal strokes or deaths from any cause (whatever occurs first) within 30 days after the intervention (either isolated CABG or synchronous CABG + CEA).
The primary effect expected from isolated or combined surgery is to reduce the rate of myocardial infarction. The clinically relevant figure, however, is the sum of periprocedural complications and ipsilateral stroke during a longer period. Isolated CABG is expected to go along with lower 30-day complication rates but higher long-term ipsilateral ischemia rates. The reverse is to be expected from the combined CABG / CEA procedure. The primary efficacy endpoint takes into account only the short-term effects. In contrast, ongoing studies will have to demonstrate which perioperative complication rate is compatible with a long-term benefit of CEA compared to modern medical treatment.

### 2.3 Assessment of safety

Safety is assessed as the rate of any nonfatal stroke or death from any cause within 30 days after CABG. This safety endpoint has been used in many trials concerned with treatment of carotid stenoses.[9, 26, 27] In addition, there will be a special focus on complications and serious adverse events within 30 days after CABG. The etiology of death within 30 days after CABG therefore should be verified by autopsy, if possible.

### 2.4 Secondary outcomes

Secondary efficacy endpoints are single components of the primary endpoint, myocardial infarction, technical failures, duration of ventilatory support, change of cognitive performance on the Demtec scale and observations at different time points. These are described in detail in chapter 6.3.

## 3 TRIAL DESIGN

CABACS is a randomized, controlled, open, multi-center group sequential trial with 2 parallel arms and blinded observers. All patients are treated with a best medical treatment (BMT) regimen tailored to their individual risk factor profile consisting in treatment of risk factors, lipid-lowering and anti-platelet medication. Rules for the BMT will be formulated by the BMT subcommittee (see Investigator site file).
The overall duration of the trial is expected to be approximately 8 years. Results of the primary endpoints are expected after 4.5 years. Recruitment of subjects will start in October 2010. The actual overall duration of recruitment may vary.
Patients will be allocated in a concealed way by central randomization before surgery.
A clinical report of each outcome event will be prepared by the ZKSE (unblinded part), which removes all information about the allocated treatment. Subsequently, this report will be presented to the three independent members of the clinical adjudication committee who have not been involved in the treatment of the patient so far and who classify the event as blinded adjudicators / blinded observers. Each adjudicator will send his / her classification to the ZKSE. If the three classifications differ, the outcome event

will be discussed by all members of the clinical adjudication committee considering the majority vote.

# 4 SELECTION OF SUBJECTS

## 4.1 Number of subjects

As calculated in section 9.1 ‘Sample Size Calculation’, 1,160 subjects should be enrolled in the clinical trial, 580 to the isolated CABG-arm and 580 to the combined CABG / CEA arm. Recruitment and treatment of subjects should be performed in around 35 - 40 trial centers. The minimum / maximum number of subjects per trial centre should be 10 / 100.

## 4.2 General criteria for subjects’ selection

In daily clinical practice most asymptomatic carotid stenoses are discovered by extracranial ultrasound techniques performed for screening purposes in patients with vascular disease in other vascular territories. Only few stenoses are diagnosed by extracranial MRA or CT-angiography. Due to the ultrasound-based screening procedure used in CABACS, the study population should closely resemble the general population with asymptomatic carotid stenoses undergoing CABG.

## 4.3 Inclusion criteria

Subjects meeting all of the following criteria will be considered for admission to the trial:

- Asymptomatic (past 180 days) stenosis ≥ 80% (following criteria of the ECST[28]) of the extracranial carotid artery in patients scheduled for CABG
- Negative pregnancy test in pre-menopausal women
- Written informed consent and full legal capacity
- Carotid stenosis treatable with CEA
- Ability of the patient to participate in follow-up examinations

## 4.4 Exclusion criteria

Subjects presenting with any of the following criteria will not be included in the trial:

- Non-atherosclerotic stenosis (e.g. dissection, floating thrombus, fibromuscular dysplasia, tumor)
- Complete occlusion of the carotid artery to be treated
- Prior stenting of the carotid artery to be treated
- Stenosis following radiotherapy
- Additional higher grade intracranial or intrathoracic stenosis (tandem stenosis)
- Recent (past 180 days) ischemic symptoms ipsilateral to carotid stenosis or occlusion
- Contralateral carotid occlusion or other known indication for carotid revascularization (apart from scheduled CABG)

- Myocardial infarction (NSTEMI or STEMI) within the past 7 days or hemodynamically unstable patients
- Known high risk for cardiogenic embolism requiring anticoagulation (mechanical heart valve, chronic atrial fibrillation, left ventricular thrombus, left ventricular aneurysm)
- Evidence for intracranial bleeding within the past 90 days
- Modified Rankin Scale score >3 or severe aphasia
- Patients unlikely to survive more than 1 year due to concomitant diseases
- Planned combined cardiac valve replacement or any other cardiac surgery beyond CABG (+/- CEA) during the procedure
- Major surgery (apart from study procedures) planned within 8 weeks from randomization
- Participation in another clinical trial

No patient will be allowed to enroll in this trial more than once.

## 4.5 Criteria for withdrawal

### 4.5.1 Withdrawal of subjects

A patient may be withdrawn from all trial related procedures (excluding follow-up visits and central follow-up by telephone) for the following reasons:

- at his/her own request or at request of his legal representative
- non-adherence to the trial-related requirements which may (have) influence(d) the validity of the trial data
- if, in the investigator's opinion, continuation of the trial would be detrimental to the patient's well-being, e.g. if a patient was randomized into the isolated CABG arm and suffers ipsilateral neurological symptoms prior to CABG, patient treatment should be based on current guidelines
- occurrence of exclusion criteria prior to CABG ± CEA.
- if, in the investigator's opinion, protocol violations caused by the patient would lead to invalid data (e.g. non-compliance with planned study procedures)

The Executive Committee (see chapter 10.2) decides about withdrawal of subjects from trial treatment in case of occurrence of criteria mentioned above.

In all cases, the reason for withdrawal must be recorded in the CRF and in the patient's medical records. In case of withdrawal of a patient at his / her own request, the reason should be asked for as extensively as possible and documented, even though the patient is entitled not to answer. All efforts will be made to follow the patient and all examinations scheduled for the final trial day will be performed as far as possible and documented for all subjects. All ongoing Complications / Serious Adverse Events (SAEs) of withdrawn subjects have to be followed-up until no more signs and symptoms can be verified and the patient is in stable condition.

### 4.5.2 Replacement of Subjects

Subjects will not be replaced.

### 4.5.3 Premature Closure of the Clinical Trial

If the 30-day complication rate (any stroke or death) of one or both treatments exceeds 10% (both point estimator and lower limit of 80% confidence interval are larger than 10%), the DSMB can give a recommendation to the steering committee to stop the trial. If the 30-day complication rate exceeds 15%, the DSMB should recommend to stop the trial early.
The trial can be prematurely closed or suspended by the Steering Committee in case of new data about the risk-benefit of one of the treatment regimes becoming available, if the p-value for the treatment effect is smaller than α 1 = 0.0052 at the first planned interim analysis, or if the DSMB recommends study closure. The Ethics Committee (EC) must then be informed. Furthermore, the Ethics Committee(s) themselves may decide to stop or suspend the trial.
Should the trial be closed prematurely, all trial material (completed, partially completed, and blank CRF, etc.) must be returned to the Center for Clinical Trials (ZKSE; Essen)
All involved investigators have to be informed immediately about a cessation /suspension of the trial. The decision is binding to all trial centers and investigators.

# 5 TRIAL PROCEDURES

## 5.1 Description of trial days

Study visits are scheduled as follows:

- Screening
- Randomization (Screening and randomization can be done on the same day)
- Surgical treatment (day 0), as soon as possible (max. 7 days) after randomization
- Day 7 ± 1 after CABG
- 30 ± 3 days after CABG (or randomization if surgical treatment was cancelled)
- 1 year ± 14 days after randomization
- 2 years ± 30 days after randomization (telephone follow-up)
- 3 years ± 30 days after randomization (telephone follow-up)
- 4 years ± 30 days after randomization (telephone follow-up)
- 5 years ± 30 days after randomization (telephone follow-up)

An overview about the procedures to be done at specific visits can be found on page 9.

## 5.2 Screening and randomization

After initiation of the center, all patients will be consecutively screened and all eligible patients who are willing to participate will be included in the trial. To qualify for this trial, patients must have met all of the above described inclusion and none of the exclusion criteria. Screening and randomization can be done on the same day. CABG ± CEA must be done as soon as possible (max. 7 days) after randomization. Events occurring between randomization and operation are counted within the allocated treatment group. Events occurring between screening and randomization are not counted.
In order to achieve comparable groups, patients will be allocated in a concealed way by central randomization ≥1 day before surgery. To address “concealment of allocation”,

the randomization will be done centrally by the Center for Clinical Trials, Essen ("Zentrum für Klinische Studien Essen", ZKSE; www.zkse.de) and each patient who is registered and randomized will be part of the ITT analysis set. The randomization list will be kept in safe and confidential custody at the ZKSE.
Before randomization, the following procedures (5.2.1- 5.2.6) have to be done:

### 5.2.1 Informed consent

The patient must be able to understand the nature of the trial and the related procedures and sign and date the informed consent form in person. Informed consent must be available before any study related procedures.

### 5.2.2 Risk factor screening

- Hypertension (yes / no)
- Diabetes (yes / no)
- Hyperlipidemia (yes / no)
- Smoking history
- Bodyweight, height

### 5.2.3 Neurological examination

Modified Rankin Scale (mRS) and NIH-Stroke Scale (NIHSS) are determined at screening as the basis for the follow-up-examinations. A mRS > 3 at baseline is an exclusion criterion. All neurologists should have a NIHSS certificate (http://asa.trainingcampus.net/uas/modules/trees/windex.aspx).

### 5.2.4 Prior and concomitant illnesses

Relevant additional illnesses present at the time of informed consent are regarded as concomitant illnesses and will be documented on the appropriate pages of the case report form (CRF).
Patients with recent (last 180 days) ischemic symptoms ipsilateral to the territory of the vessel to be treated are not allowed to be included into the trial.

### 5.2.5 Prior and concomitant treatments

Relevant concomitant therapies (e.g. antiplatelets, antihypertensives, lipid-lowering-drugs) will be documented preoperatively, at 30 days, 1 year and thereafter yearly up to five years after CABG.
If another major surgery (apart from CABG ± CEA) is planned during the first 8 weeks after randomization patients should not be included prior to this operation.

### 5.2.6 Ultrasound examination

The ultrasound examination including color-coded extracranial Duplex and examination of the intracranial circulation should be done by an experienced examiner (preferably with either DGKN or DEGUM ultrasound certificate). Documentation contains at least grading of the stenosis, plaque morphology and a measurement of the Intima-Media-Thickness in the common carotid arteries. Stenosis severity as measured with ultrasound is usually expressed following the method of the ECST. If a conversion from a NASCET measurement is necessary, the following equation will be used: %NASCET

= (%ECST - 43) x (100 / 57).[29] Grading of stenosis by CT angiography or MR angiography needs to be confirmed by ultrasound examination.

## 5.3 Treatment

### 5.3.1 Allocation to treatment / Randomization

Eligibility of a patient will be determined by a study neurologist. A study surgeon needs to confirm that treatment is feasible. CABG with or without CEA must be performed as soon as possible (max. within 7 days) after randomization. In the case of a bilateral stenosis, randomization is performed only once, usually for the side with the higher grade stenosis.
For this open trial, a centralized randomization will be set up. All eligible patients will be registered and randomized to achieve balanced prognostic factor distributions for the factors age (< 60 years or ≥ 60 years), sex (male or female), modified Rankin Score (0-1 or 2-3) and the factor centre.

The completed CRF Part Randomization is to be sent by fax to the

**Center for Clinical Trials Essen (ZKSE), University of Duisburg-Essen**
**Hufelandstr. 55, 45147 Essen**
**Fax: 0201 723 5933, Phone: 0201 723 4134**

Documentation of all potentially eligible patients in a screening log is mandatory including reasons for non-inclusion into the trial. Inclusion of all patients meeting all inclusion criteria and none of the exclusion criteria is mandatory. Non-compliance with this rule may be a reason for exclusion of a site. For further details, e.g. concealment of allocation see 5.2.

### 5.3.2 Best medical treatment

All patients will be treated with up-to-date medication following national and international guidelines. Recommendations for this treatment will be given by the 'Best Medical Treatment' subcommittee (see TMF and investigator site file).

### 5.3.3 Isolated CABG without carotid endarterectomy

Standards for surgical treatment are formulated by the Surgical quality subcommittee (see TMF and investigator site file)

### 5.3.4 Combined CABG and carotid endarterectomy

Standards for surgical treatment are formulated by the Surgical quality subcommittee (see TMF and investigator site file)

## 5.4 Blinding

Due to the nature of the treatments involved, blinded treatment is not possible. On ultrasound upon follow-up, it is also obvious how the patient was treated. Therefore, the

study has to follow an open design. To minimize a potential bias, the neurologist who is not directly involved in the operation is responsible for the follow-up examinations and end-point assessment. For endpoint adjudication, no information on treatment group will be made available (blinded observers).

### 5.5 Follow-up examinations

The duration of the trial for each patient is 5 years. After the periprocedural phase and a follow-up visit after one year, yearly telephone follow-ups are scheduled. Each visit consists of a neurological examination, an event screening, a risk factor screening, and an ultrasound examination. Telephone follow-ups consist of an event screening only.
Events are screened using a standardized questionnaire. An event is suspected if one of the following questions has been answered positively:

- "Ist (seit der letzten Untersuchung) eine (vorübergehende) Sehstörung auf einem Auge aufgetreten?"
- „Ist (seit der letzten Untersuchung) eine Schwäche oder Empfindungsstörung auf einer Körperseite aufgetreten?"
- „Ist (seit der letzten Untersuchung) eine Sprachstörung aufgetreten?"
- „Haben sich (seit der letzten Untersuchung) neue Beschwerden entwickelt?"

If a cerebrovascular event is suspected, appropriate information from the treating physician should be obtained. During the first year, an appropriate neuroimaging should be performed in case of a suspected cerebrovascular event to distinguish between possible types of stroke. In case of an ipsilateral cerebrovascular event in a patient randomized into the isolated CABG arm, the patient should be considered for CEA or carotid artery stenting following current guidelines for treatment of symptomatic stenosis.
Risk factor screening, assessment of medication, and ultrasound examinations have to be done as described above (section 5.2)
In addition, the mRS and NIHSS have to be assessed and documented after 30 days and 1 year.

### 5.6 Plan for medical treatment and care after the trial

Medical treatment with risk factor modification will be continued after the end of the follow-up period (5 years in each individual). The general practitioner of the patient will be responsible for control of these risk factors and antiplatelet therapy.

## 6 ASSESSMENTS

### 6.1 Definitions

Ischemic Stroke: Defined by at least one of the following criteria:

- Sudden onset of focal neurological symptoms lasting more than 24 hours, with no apparent cause other than cerebral ischaemia.
- Sudden onset of focal neurological symptoms with the presence of cerebral infarction in the appropriate territory on brain imaging (CT or MRI), regardless of the duration of symptoms (less than or more than 24 hours).

Hemorrhagic Stroke:

Sudden onset of focal neurological symptoms with the presence of cerebral haemorrhage in the appropriate territory on brain imaging (CT or MRI), regardless of the duration of symptoms (less than or more than 24 hours) and regardless of the cause of the haemorrhage (spontaneous or secondary to trauma, tumour or another cause).

Unknown type of stroke:

In some cases, the type of stroke cannot be determined with certainty, particularly when brain imaging or an autopsy report or an operation report cannot be obtained, despite all efforts. This type of event will be classified as unknown type of stroke, if the symptoms last more than 24 hours.
Ipsilateral stroke: Stroke within the territory of the treated vessel or higher grade stenotic carotid artery at screening.
Disabling stroke: Stroke leading to a disability of at least 4 on the modified Rankin scale at day 30 after symptom onset.
Myocardial infarction: Any one of the following criteria satisfies the diagnosis for an **acute MI** [30]
1. Detection of rise and/or fall of cardiac biomarkers (preferably troponin) with at least one value above the 99$^{th}$ percentile of the upper reference limit (URL) together with evidence of myocardial ischemia with at least one of the following

- Symptoms of ischemia
- ECG changes indicative of new ischemia (new ST-T changes or new left bundle branch block [LBBB])
- Development of new pathological Q waves on the ECG
- Imaging evidence of a new loss of viable myocardium or new regional wall motion abnormality

2. Pathological findings of an acute MI.
3. In patients who have undergone CABG (< 72h) with normal baseline troponin values: Elevation of biomarkers greater than 5 times the 99$^{th}$ percentile URL together with either

- New pathological Q waves
- New left bundle branch block
- Angiographically documented new graft or native coronary artery occlusion
- Imaging evidence of a new loss of viable myocardium

Vascular death:

- death related to a cardiac or vascular cause.
- death due to haemorrhage.
- death due to pulmonary embolism.
- sudden death: death occurring in less than 24 hours, unexpectedly in a subject in apparent good health and whose condition was stable or was improving.
- death with no documented non-vascular cause.
- fatal stroke: death occurring within 30 days of a stroke (ischaemic or haemorrhagic).

Non-vascular death:
Death due to a documented non-vascular cause (infection, cancer, accident, suicide, etc.).
Technical failure: Inability to treat the stenosis with CEA or residual stenosis after CEA of at least 70% or occlusion following ultrasound-criteria at day 30.

Restenosis: Recurrent stenosis of at least 70% or occlusion following ultrasound-criteria. Observation period: 30 day endpoints for patients randomized into either group are evaluated after 30±3 days after the intervention (either isolated CABG and combined CABG + CEA). The long-term (up to 5 years) observational period starts after the intervention (either isolated CABG or combined CABG + CEA), annual assessment can be done in a time period ±14 days around the calculated date (±30 days from the second year on).

## 6.2 Evaluation of safety

Safety is assessed as the rate of any stroke within 30 days of treatment or death from any cause within 30 days after CABG+/- CEA.

## 6.3 Evaluation of efficacy

### 6.3.1 Primary endpoint

The primary efficacy endpoint is the event rate of nonfatal strokes or deaths from any cause (whatever occurs first) within 30 days after the intervention (either isolated CABG or synchronous CABG + CEA).

### 6.3.2 Secondary endpoints

- Number of ischemic strokes ipsilateral to the initially higher grade, not occluded stenotic carotid artery within 30 days and 1 year
- Any stroke or vascular death within 30 days, 1 year and 5 years
- Deaths from any cause within 30 days, 1 year and 5 years
- Number of disabling strokes (definition: stroke with resulting impairment >3 on the modified Rankin Scale) within 30 days and 1 year
- Change of cognitive performance on the Demtec scale from randomization to 30 days and 1 year
- Technical failure of intervention
- Number of myocardial infarctions within 30 days, and from 30 days to 1 year
- Duration of ventilatory support after operation (CABG ± CEA)

### 6.3.3 Additional analysis

- Total length of hospital stay and G-DRG for acute hospital stay.
- Total length of ICU stay

## 6.4 Endpoint evaluation

All suspected strokes, as well as all myocardial infarctions, and all deaths will be evaluated by a committee of experts blinded to treatment, who will determine the final classification of these events. This committee, named ‘clinical adjudication committee’ is described in section 10.3.

# 7 COMPLICATIONS AND SERIOUS ADVERSE EVENT REPORTING

## 7.1 Definitions

### 7.1.1 Complications

Only surgical complications will be documented. This includes the following complications:

| | Documentation of complication on | | |
|---|---|---|---|
| | Endpoint form | BQS form | CRF |
| Bleeding requiring reoperation | | x | |
| Deep wound infection | | | X |
| Sepsis | | | X |
| Deep vein Thrombosis | | | X |
| Pulmonary embolism | | | X |
| Pneumonia | | | X |
| Stroke (Ischemic, Hemorrhagic, Disabling, Ipsilateral) | X | X | |
| Cervical nerve injury | | | X |
| Re-Thoracotomy | | X | |
| Re-CEA | | | X |
| Myocardial infarction | X | X | |
| Severe hemodynamic instability > 24 h | | | X |
| Carotid restenosis/occlusion | X | | |
| Carotid dilatation | | | X |
| Cardiopulmonary resuscitation | | | X |
| Others | | X | X |

In the ICU situation there is a substantial background noise of signs and symptoms. If the documentation had to be done according to the GCP Adverse events definition, the physicians would have to document more than 100 AE per patients. This is not practicable. A method to reduce the effect of the background noise is to document the complications according to BQS and only those other complications with a potential relationship to surgery (CABG +/- CEA).

A pre-existing disease or symptom will not be considered a complication unless there will be an untoward change in its intensity, frequency or quality and this change has a

potential relationship to surgery (CABG +/- CEA). This change will be documented by an investigator.
Surgical procedures themselves are not complications; they are therapeutic measures for conditions that require surgery. The condition for which the surgery is required may be a complication. Planned surgical measures permitted by the clinical trial protocol and the condition(s) leading to these measures are no complications, if the condition leading to the measures was present prior to inclusion into the trial.

### 7.1.2 Serious adverse event

All serious adverse events (irrespective of a possible causal relationship to the surgery) will be documented during the first 30 days after surgery only.
A serious adverse event (SAE) is one that:

- results in death
- is life-threatening (the term life-threatening refers to an event in which the patient was at risk of death at the time of event and not to an event which hypothetically might have caused death if it was more severe)
- requires patient hospitalization or prolongation of existing hospitalization
- results in persistent or significant disability / incapacity or
- is a congenital anomaly / birth defect.
- requires ventilatory support beyond 30 days after CABG +/- CEA (if not documented already as SAE (e.g. prolongation of hospitalization))

## 7.2 Period of observation and documentation

Only surgical complications reported by the patient or detected by the investigator will be collected during the trial and must be documented on the appropriate pages of the CRF / BQS forms. Complications must also be documented in the patient's medical records.
In this trial, all complications that occur during 30 days after the operation (CABG ± CEA) will be documented on the pages provided in the CRF / BQS forms.

## 7.3 Reporting of SAEs / OE by investigator

All serious adverse events or outcome events must be reported by the investigator to the ZKSE within 7 days using the 'Serious Adverse Event / Outcome event' form.

**Center for Clinical Trials Essen (ZKSE), CABACS Trial**
**University of Duisburg-Essen, Hufelandstr. 55, 45147 Essen**
**Fax: 0201 723 947 4134, Phone: 0201 723 4134**

Forms are in the investigator site file. The report must be as complete as possible.
A report of an outcome event should include details concerning the type of stroke (ischemic vs. hemorrhagic, territory of stroke, and severity of stroke) or cause of death.
All OEs are also evaluated by the CAC.

### 7.4 Expedited reporting

According to the Declaration of Helsinki every investigator should report all SAE to his Ethics Committee. In this trial with up to 40 sites, this reporting is counterproductive. A central reporting will improve the safety of all trial patients. With the Ethics Committee in Essen the following agreement was concluded:
A report (analogous to the annual safety report) will be sent to the independent Ethics Committee (University of Duisburg-Essen) and the sites. This report includes all SAE and OE. This report will be sent every 3 months (4 times a year).
SAEs resulting in death are to be reported separately to the Ethics Committee (University of Duisburg-Essen) within 14 days.
These assessments will be done by the Trial coordinator Dr. med. Stephan Knipp, Dept. of Thoracic and Cardiovascular Surgery; University of Duisburg-Essen.

## 8 DATA MANAGEMENT

### 8.1 Data collection

The sponsor will provide blank CRF to the sites. Each CRF page has a unique identifier. This is the patient number.
All findings will be documented in the patient's medical record and in the CRF. The investigator is responsible for ensuring that all sections of the CRF are completed correctly and that entries can be verified against source data. Any errors should have a single line drawn through them so that the original entry remains legible and the correct data should be entered at the side with the investigator's signature, date and reason for change. Self-explanatory corrections need not to be justified.
The correctness of entries in the CRF will be confirmed by dated signature of the responsible investigator. After finalization of the visit after 30 days and 1 year, the original CRF will be copied for local safekeeping and the originals are transferred once a month to the data management of ZKSE.

**Center for Clinical Trials Essen (ZKSE) c/o IMIBE**
**CABACS Trial, Frau Anja Marr**
**University of Duisburg-Essen, Hufelandstr. 55, 45147 Essen**

The copies of the CRF will be kept by the investigator.

### 8.2 Data handling

Data handling is described in detail in the Data Management Plan and the Data Validation Plan.
To ensure data quality, a double data entry will be done. After completion of data entry, checks for plausibility, consistency, and completeness of the data will be performed. Based on these checks, queries will be produced combined with the queries generated

by visual control. A responsible investigator will be obliged either to correct the implausible data or to confirm its authenticity and to give appropriate explanation.
Further checks for plausibility, consistency, and completeness of the data will be performed after completion of the study. Queries will be generated on the basis of these checks combined with a visual control by a responsible monitor/data manager.
All missing data or inconsistencies will be reported back to the sites and clarified by the responsible investigator. If no further corrections are to be made in the database, it will be declared closed and used for statistical analysis.
All data management activities will be done according to the current Standard Operating Procedures (SOPs) of the ZKSE.
To ensure the safety of participants in the study, and to ensure accurate, complete, and reliable data, the investigator must keep records of laboratory tests, clinical notes, and patient medical records in the patient files as original source documents for the study.

### 8.3 Storage and archiving of data

All important trial documents (e.g. original CRF) will be archived for at least 10 years after the end of the trial.
The investigator(s) will archive all trial data (source data and Investigator Site File (ISF) including patient identification list, signed informed consent forms and relevant correspondence) according to the section 4.9 of the ICH Consolidated Guideline on GCP (E6).

## 9 DATA ANALYSIS / STATISTICAL METHODS

### 9.1 Sample size calculation

Sample size calculations were performed for the primary efficacy endpoint. The sample size calculation is based on using the Brownian motion approximation to a 2x2 $X^2$ test (two-sided analysis) using the classical boundaries of O'Brien and Fleming (1979). Note that the maximum sample size of the group sequential plan is slightly larger than the sample size for the fixed sample size case which can e.g. be performed for the $X^2$ test using nQuery Advisor ® 6.0. The average sample size of the sequential plan is smaller than the sample size for the fixed sample size case. The prior assumptions are a frequency of 8.5% in the control group (synchronous CABG and carotid endarterectomy), and 4% in the experimental group (CABG without carotid endarterectomy) based on data from the literature.[7, 17] To detect this difference with a total α=5%, a maximum sample size of n = 2 x 550 = 1,100 patients with complete information for the primary endpoint will lead to a power of 84%. Note that intra-surgeon correlation of the primary outcome may lead to an underestimation of the statistical power which is why a power >80% was chosen. The table below shows the impact on the power for five alternative scenarios (rates differing from those expected but ignoring intra-surgeon correlation) for n = 1,100 patients. In order to address a potential drop out rate of 5% overall (based on empirical data), another 60 patients have to be randomized. Thus, the total maximum sample size is n = 2 x 580 = 1,160 patients that have to be allocated to the treatment arms (scenario 1). With this sample size, the power of all scenarios is larger than 65% and except for scenario 3 also larger than 80%.

**Table 2:** Power considerations for the CABACS trial.

| | | scenario | | | | | |
|---|---|---|---|---|---|---|---|
| | | 1 | 2 | 3 | 4 | 5 | 6 |
| rate of (first) nonfatal stroke or death within 30 days after CABG [%] | control group: **simultaneous** CABG and carotid endarterectomy | 8.5 | 9.5 | 7.5 | 8.5 | 9.5 | 7.5 |
| | experimental group: CABG **without** carotid endarterectomy | 4 | 4 | 4 | 3 | 3 | 3 |
| power [%]# of sequential plan (n=1,100) | | 84 | 95 | 66 | 97 | 99 | 90 |
| power [%]# of sequential plan (n=1,160 - i.e. in case of no drop-outs; interim analyses after 550 patients randomized and followed-up for 30 days) | | 87 | 96 | 69 | 98 | 99 | 92 |

#based on simulations using 10,000 replicates and a two-sided 2x2 $X^2$ test with $\alpha_1 = 0.0052$ and $\alpha_2 = 0.0480$ for testing

## 9.2 Definition of trial population to be analyzed

The primary confirmatory analysis will be performed on the intention-to-treat (ITT) population which includes all patients randomized and treated (full analysis set). In addition, a per-protocol (PP) analysis will also be performed after exclusion of patients who did not undergo the allocated operation, or other serious protocol violations. The following are considered major protocol deviations:

- Not meeting one or more of the Inclusion Criteria and/or falling into one or more of the Exclusion Criteria
- Not finishing the allocated therapy (change of treatment group)
- Operation done by a non-certified interventionalist or using noncertified material
- Endpoint event between randomization and surgical treatment

In case of problems with the decision if a protocol violation is major, the blinded Clinical Adjudication Committee is responsible for the allocation.

## 9.3 Statistical methods

**Safety and Efficacy**: The analysis of the primary outcome will be a comparison of the experimental group (CABG **without** CEA; $\pi_{experimental}$) with the control group (**synchronous** CABG and CEA; $\pi_{control}$) with regard to differences in first nonfatal stroke or death within 30 days after CABG/CEA. In particular, the null ($H_0$) and the alternative hypotheses ($H_1$) are:

$H_0$: $\pi_{experimental} = \pi_{control}$
$H_1$: $\pi_{experimental}$ # $\pi_{control}$

where $\pi_.$ denotes the event rate for each group. Note that we consider a two-sided test with α=0.05 as both superiority of the experimental or superiority of the control group are of interest. The confirmatory analysis on the intention-to-treat analysis set (see above) will be performed by a generalized linear mixed effects model[31] including the prognostic factors of the randomization as covariates (fixed factors: age: < 60 years or ≥ 60 years;

sex: male or female; modified Rankin Score: 0-1 or 2-3; random factor: centre). The null hypothesis can be rejected if the p-value related to the Wald test statistic for the treatment effect is smaller than either $\alpha_1$ = 0.0052 at the first planned interim analysis (after 550 patients are randomized and followed-up for 30 days) or smaller than $\alpha_2$ = 0.0480 at the final analysis of all data (O'Brien and Fleming group sequential plan) such that the total α is 0.05 (two-sided). In addition to the sequential multiple testing, no other multiple testing issues arise for the confirmatory analysis.
Nonetheless, we will perform sensitivity analysis of the primary outcome. These include per-protocol analyses, subgroup analyses stratified by gender and worst/best case scenario analyses in case of missing data of the primary efficacy endpoint.

Details on the biometric analyses will be defined in the statistical analysis plan which has to be authorized by the biometrician, the sponsor, and the LKP.

### 9.4 Interim analyses

The trial is designed as a group sequential plan with one planned interim analysis. The null hypothesis can be rejected if the p-value related to the Wald test statistic for the treatment effect is smaller than $\alpha_1$ = 0.0052 at the first planned interim analysis (after 550 patients are randomized and followed-up for 30 days; O'Brien and Fleming group sequential plan).

## 10 STUDY ADMINISTRATION

All persons responsible for the study are listed in the investigator site file including name, address, phone and email.
In order to monitor specific aspects of the current trial, the following Reference Committees will be established: Data Safety and Monitoring Board (DSMB), Steering Committee (SC), Executive Committee (ExC) and a Clinical Adjudication Committee (CAC). The work of these committees will be based on the 'Guideline on Data Monitoring Committees' EMEA/CHMP/EWP/5872/03

### 10.1 Data Safety and Monitoring Board (DSMB)

An independent Data Safety and Monitoring Board (DSMB) has been assembled. The DSMB is composed of independent experts in the field of Neurology, Vascular Surgery and Cardiovascular Surgery, Cardiology, and Biometry who assess the progress, safety of data and critical efficacy endpoints. The mission of the DSMB is to ensure the ethical conduct of the trial and to protect the safety interests of patients in this trial. In the context of overall patient safety the DSMB will receive periodic reports (also including center specific information) as well as any special reports as requested by the DSMB and to be prepared by the trial data center. The DSMB will have access to all trial data. The need and frequency of face-to-face meetings will be determined by the DSMB and the Steering Committee, taking into account the possibility of teleconferencing and other electronic conference options. DSMB safety data review meetings will regularly be held twice a year, with the first one scheduled 8 weeks after the first **200** patients have passed **30** days after therapy. Another meeting will be held after the interim analysis

after 550 patients. The last meeting will be scheduled when all patients have passed this time limit.
The DSMB will define details of their working procedures in an extra protocol to keep the independence from the study. The DSMB protocol will be part of the TMF.

## 10.2 Steering Committee (SC) and Executive Committee (ExC)

The SC consists of members of all involved disciplines that actively take part in the execution of this study. Because of the size of the steering committee, 3 members of the steering committee build an Executive Committee (ExC)

## 10.3 Clinical Adjudication committee (CAC)

A blinded clinical adjudication committee will confirm all outcome event notices, as it will make the final decision on whether a patient had any outcome event. All patients thought to have an outcome event will have their CRF and, if necessary, additional information examined by the members of this committee. The committee can also request additional ancillary information from an individual study investigator to assist their review. The CAC will also confirm the type (e.g. ischemic, hemorrhagic) and localization of each stroke or cause of death.

## 10.4 Quality Subcommittees

Two subcommittees with experts in this field have formulated rules and recommendations for diagnosis and treatment. There are recommendations for best medical treatment and cardiovascular surgery. These recommendations are appendices of this protocol. The subcommittees have also defined details of the quality criteria (see section 10.5.2) for participation of an investigator. The head of each sub-committee is also a member of the SC.

## 10.5 Control of treatment quality

### 10.5.1 Centers

Each study center must consist of at least one neurologist and one cardiovascular / vascular surgeon. Certification of a participating center will follow upon compliance to all quality criteria. The steering committee can withdraw the certification to participate in the trial on recommendation of the DSMB and/or the monitoring agency.

### 10.5.2 Investigators

For all involved disciplines quality criteria are defined by the appropriate quality committee (see TMF). Every neurologist has to demonstrate his/her ultrasound-expertise and should have a NIHSS-certificate. Cardiovascular / vascular surgeons must demonstrate their experience as defined in the appendix of the surgical quality subcommittee. The local principal investigator has to confirm this documentation. Together with the DSMB the quality committees will monitor local complication rates and can recommend the steering-committee or the executive committee to withdraw a certificate.

### 10.6 Investigator Site File

The investigator site file will contain the following documents
- Declaration of Helsinki
- Study Protocol
- CRF
- Informed consent forms
- SAE / OE Form
- Monitoring log
- Patient identification list
- Approval of the Ethics Committee
- Standard procedures of the ‘Best Medical Treatment’ subcommittee
- Standard procedures of the Surgical quality subcommittee
- Qualification of investigators
- List of authorized persons (including study nurses) entitled to document in the CRF

All persons responsible for the study are listed in the investigator site file including name, address, phone and email.
- Participating sites.
- Quality Subcommittees
- Steering Committee (SC) and Executive Committee (ExC)
- Clinical Adjudication committee (CAC)

## 11 ETHICAL AND LEGAL ASPECTS

### 11.1 Good Clinical Practice

The procedures set out in this trial protocol, pertaining to the conduct, evaluation, and documentation of this trial, are designed to ensure that all persons involved in the trial abide by Good Clinical Practice (GCP) and the ethical principles described in the applicable version of the Declaration of Helsinki and the “Berufsordnung für Ärzte”. The trial will be carried out in keeping with local legal and regulatory requirements.

### 11.2 Patient information and informed consent

Before being admitted to the clinical trial, the patient must consent to participate after the nature, scope, and possible consequences of the clinical trial have been explained in a form understandable to him or her. The patient and investigator must sign and date the informed consent. The signed Informed Consent Form will be filed by the investigator.
A copy of the signed informed consent document must be given to the patient

### 11.3 Patient insurance

No patient or travel accident insurance will be taken out. Every hospital or surgeon is required to hold their own insurance so patients will be adequately covered in case of

malpractice. Although desirable, no insurance can cover the risk inherent to either study procedure which moreover both constitute the current standard of care.

### 11.4 Confidentiality

The data obtained in the course of the trial will be treated pursuant to the Federal Data Protection Law (Bundesdatenschutzgesetz, BDSG).
To enable the 5 year follow-up, the contact data of the patient will be archived on paper in the clinical project management unit. These data include patient number, name, address, (cell) phone, email, contact data of the family doctor and, if possible, contact data of a family member. The permission to store these data in a database strictly kept separate from the clinical data will be obtained in the informed consent.
Other clinical data of the patients will be identified solely by means of their date of birth (month and year only), and an individual identification code (patient number). Trial findings stored on a computer will be stored in accordance with local data protection law and will be handled in strictest confidence. For protection of these data, organizational procedures are implemented to prevent distribution of data to unauthorized persons. The appropriate regulations of local data legislation will be fulfilled in its entirety.
The patient consents in writing to release the investigator from his/her professional discretion in so far as to allow inspection of original data for monitoring purposes by health authorities and authorized persons (inspectors, monitors, auditors). Authorized persons (clinical monitors, auditors, inspectors) may inspect the patient-related data collected during the trial ensuring the data protection law.
The investigator will maintain a patient identification list (patient numbers with the corresponding patient names) to enable records to be identified.

### 11.5 Responsibilities of investigator

The principal investigator should ensure that all persons assisting with the trial are adequately informed about the protocol, any amendments to the protocol, the trial treatments, and their trial-related duties and functions.
The principal investigator should maintain a list of subinvestigators and other appropriately qualified persons to whom he or she has delegated significant trial-related duties.

### 11.6 Approval of trial protocol and amendments

Before the start of the trial, the trial protocol, informed consent document, and any other appropriate documents have been submitted to the independent Ethics Committee (EC) in Essen. A written favourable vote of each EC is a prerequisite for initiation of this clinical trial. The statement of EC should contain the title of the trial, the trial code, the trial site, and a list of reviewed documents. It must mention the date on which the decision was made and must be officially signed by a committee member. This documentation must also include a list of members of the EC present on the applicable EC meeting and a GCP compliance statement.
For each site a separate approval must be obtained.

Before the first patient is enrolled in the trial, all ethical and legal requirements must be met. All planned substantial changes will be submitted to ECs in writing as protocol amendments. They have to be approved by the ECs.

### 11.7 Continuous information to independent ethics committee

The EC will be informed in case the risk / benefit assessment changes or any other new and significant hazards for subjects' safety or welfare occur. Furthermore, a report on all observed serious adverse events (SAEs) will be submitted four times a year. The EC will be informed at the end of the trial.
Application for registration of the trial will be filed to http://www.controlled-trials.com. The protocol of the study will be published in a peer-reviewed journal.

## 12 QUALITY ASSURANCE

### 12.1 Monitoring

The ZKSE will make periodic visits to the trial site (minimum once a year, about 4-5 visits per site total). During these visits, they check periodically a random sample of patient data recorded against source (SDV). During the on-site monitoring visits a review of source documents, informed consent, primary outcome, recruitment, SAE documentation and reporting of adverse events will be done. CRFs and queries will be discussed with the investigator.
If the data quality and the protocol compliance in the trial site is good or sufficient, the monitor will visit this centre according to schedule (one visit per year). If data quality and/or protocol compliance is poor, the number of visits and/or the patient number for SDV will be increased.
This monitoring concept is in compliance with ICH GCP. Monitoring of clinical trials is often interpreted as requiring intensive on-site monitoring, but the following paragraph should be noted: "The determination of the extent and nature of monitoring should be based on considerations such as the objective, purpose, design, complexity, blinding, size and endpoints of the trial. In general there is a need for on-site monitoring, before, during and after the trial; however, central monitoring in conjunction with procedures such as investigators' training and meetings and extensive written guidance can assure appropriate conduct of the trial in accordance with GCP. Statistically controlled sampling may be an acceptable method for selecting the data to be verified." (ICH GCP 5.18.3) In this study central monitoring will be combined with on-site monitoring. The following issues will be implemented for this study:

1) In addition to the regular on-site visits, the site will be contacted through letters, web conferences, web seminars or telephone calls by the ZKSE to review study progress, investigator and patient compliance with requirements, and follow up on any issues to be addressed.
2) A central monitoring will be carried out including the following checks:
    - data collected are consistent with adherence to the trial protocol
    - no key data are missing
    - data appear to be valid (for example, range and consistency checks)
    - review of recruitment rates, withdrawals and losses to follow-up

3) On-site monitoring including SDV for a random sample of patients
4) Increasing the monitoring frequencies in trial sites with poor quality
5) In this large multi-centre study a further central monitoring of data using statistical techniques will be carried out. This statistical monitoring is useful for identifying unusual patterns of data, and can be used to detect sites or individuals where there may be deviations from the protocol.
6) ZKSE may audit the study at any time. Investigators will be given notice before an audit occurs. They are obliged to permit these visits, assist visiting study personnel and auditors as necessary and make available any records required.

### 12.2 Inspections / Audits

DFG and an auditor authorized by the sponsor may request access to all source documents, CRF, and other trial documentation. Direct access to these documents must be guaranteed by the investigator who must provide support at all times for these activities.

## 13 AGREEMENTS

### 13.1 Financing of the trial

The trial will be financed using funds of the DFG (WE 2585/3-1).

### 13.2 Publication

All information concerning the trial is confidential before publication. The data on safety and on the primary endpoint (30-day) results will be published first after the end of the trial. Publications will be prepared from a writing committee in the name of all CABACS investigators including the responsible trial statistician.

## 14 SIGNATURES

The present trial protocol was subject to critical review and has been approved in the present version by the undersigning persons. The information contained is consistent with:

- the current risk-benefit assessment of the investigational procedure
- the moral, ethical, and scientific principles governing clinical research as set out in the applicable version of Declaration of Helsinki and the principles of GCP.

The investigator will be supplied with details of any significant or new finding including complications relating to treatment.

Date: ____________ Signature: ________________________________

Name (block letters):

________________________________

Function: Principal Investigator

Date: ____________ Signature: ________________________________

Name (block letters):

________________________________

Function: Trial Coordinator

Date: ____________ Signature: ________________________________

Name (block letters):

________________________________

Function: Trial Statistician

## 15 DECLARATION OF INVESTIGATOR

I have read the above trial protocol and confirm that it contains all information to conduct the clinical trial. I pledge to conduct the clinical trial according to the protocol.
I will enroll the first patient only after all ethical and regulatory requirements are fulfilled. I pledge to obtain written consent for trial participation from all subjects.
I know the requirements for accurate notification of serious adverse events or outcome events and I pledge to document and notify such events as described in the protocol.
I pledge to retain all trial-related documents and source data as described. I will provide a Curriculum Vitae (CV) before trial start. I agree that the CV may be submitted to the EC.

Trial Center (address): ______________________________

______________________________

______________________________

Date: ___________ Signature: ______________________________

Name (block letters): ______________________________

Function: Investigator

Date: ___________ Signature: ______________________________

Name (block letters): ______________________________

Function: Investigator

Date: ___________ Signature: ______________________________

Name (block letters): ______________________________

Function: Investigator

# 16 REFERENCES


1. Bruckenberger E. Herzbericht 2007 mit transplantationschirurgie. 20. Bericht. *Hannover: Eigenverlag* 2007
2. Nalysnyk L, Fahrbach K, Reynolds MW, Zhao SZ, Ross S. Adverse events in coronary artery bypass graft (cabg) trials: A systematic review and analysis. *Heart*. 2003;89:767-772
3. Naylor R, Cuffe RL, Rothwell PM, Loftus IM, Bell PR. A systematic review of outcome following synchronous carotid endarterectomy and coronary artery bypass: Influence of surgical and patient variables. *Eur J Vasc Endovasc Surg*. 2003;26:230-241
4. Naylor AR, Mehta Z, Rothwell PM, Bell PR. Carotid artery disease and stroke during coronary artery bypass: A critical review of the literature. *Eur J Vasc Endovasc Surg*. 2002;23:283-294
5. Li Y, Walicki D, Mathiesen C, Jenny D, Li Q, Isayev Y, Reed JF, 3rd, Castaldo JE. Strokes after cardiac surgery and relationship to carotid stenosis. *Arch Neurol*. 2009;66:1091-1096
6. Naylor AR. Does the risk of post-cabg stroke merit staged or synchronous reconstruction in patients with symptomatic or asymptomatic carotid disease? *J Cardiovasc Surg (Torino)*. 2009;50:71-81
7. Schoof J, Lubahn W, Baeumer M, Kross R, Wallesch CW, Kozian A, Huth C, Goertler M. Impaired cerebral autoregulation distal to carotid stenosis/occlusion is associated with increased risk of stroke at cardiac surgery with cardiopulmonary bypass. *J Thorac Cardiovasc Surg*. 2007;134:690-696
8. Chambers BR, Donnan GA. Carotid endarterectomy for asymptomatic carotid stenosis. *Cochrane Database Syst Rev*. 2005:CD001923
9. Halliday A, Mansfield A, Marro J, Peto C, Peto R, Potter J, Thomas D. Prevention of disabling and fatal strokes by successful carotid endarterectomy in patients without recent neurological symptoms: Randomised controlled trial. *Lancet*. 2004;363:1491-1502
10. Endarterectomy for asymptomatic carotid artery stenosis. Executive committee for the asymptomatic carotid atherosclerosis study. *Jama*. 1995;273:1421-1428
11. Barner HB. Operative treatment of coronary atherosclerosis. *Ann Thorac Surg*. 2008;85:1473-1482
12. Comparison of coronary bypass surgery with angioplasty in patients with multivessel disease. The bypass angioplasty revascularization investigation (bari) investigators. *N Engl J Med*. 1996;335:217-225
13. Hoffman SN, TenBrook JA, Wolf MP, Pauker SG, Salem DN, Wong JB. A meta-analysis of randomized controlled trials comparing coronary artery bypass graft with percutaneous transluminal coronary angioplasty: One- to eight-year outcomes. *J Am Coll Cardiol*. 2003;41:1293-1304
14. Hannan EL, Racz MJ, Walford G, Jones RH, Ryan TJ, Bennett E, Culliford AT, Isom OW, Gold JP, Rose EA. Long-term outcomes of coronary-artery bypass grafting versus stent implantation. *N Engl J Med*. 2005;352:2174-2183
15. Serruys PW, Morice MC, Kappetein AP, Colombo A, Holmes DR, Mack MJ, Stahle E, Feldman TE, van den Brand M, Bass EJ, Van Dyck N, Leadley K, Dawkins

KD, Mohr FW. Percutaneous coronary intervention versus coronary-artery bypass grafting for severe coronary artery disease. *N Engl J Med*. 2009;360:961-972
16. Mortaz Hejri S, Mostafazadeh Davani B, Sahraian MA. Carotid endarterectomy for carotid stenosis in patients selected for coronary artery bypass graft surgery. *Cochrane Database Syst Rev*. 2009:CD006074
17. Naylor AR, Mehta Z, Rothwell PM. A systematic review and meta-analysis of 30-day outcomes following staged carotid artery stenting and coronary bypass. *Eur J Vasc Endovasc Surg*. 2009;37:379-387
18. Timaran CH, Rosero EB, Smith ST, Valentine RJ, Modrall JG, Clagett GP. Trends and outcomes of concurrent carotid revascularization and coronary bypass. *J Vasc Surg*. 2008;48:355-360; discussion 360-351
19. Cywinski JB, Koch CG, Krajewski LP, Smedira N, Li L, Starr NJ. Increased risk associated with combined carotid endarterectomy and coronary artery bypass graft surgery: A propensity-matched comparison with isolated coronary artery bypass graft surgery. *J Cardiothorac Vasc Anesth*. 2006;20:796-802
20. Ricotta JJ, Wall LP, Blackstone E. The influence of concurrent carotid endarterectomy on coronary bypass: A case-controlled study. *J Vasc Surg*. 2005;41:397-401; discussion 401-392
21. Ghosh J, Murray D, Khwaja N, Murphy MO, Walker MG. The influence of asymptomatic significant carotid disease on mortality and morbidity in patients undergoing coronary artery bypass surgery. *Eur J Vasc Endovasc Surg*. 2005;29:88-90
22. Hertzer NR, Loop FD, Beven EG, O'Hara PJ, Krajewski LP. Surgical staging for simultaneous coronary and carotid disease: A study including prospective randomization. *J Vasc Surg*. 1989;9:455-463
23. Dubinsky RM, Lai SM. Mortality from combined carotid endarterectomy and coronary artery bypass surgery in the us. *Neurology*. 2007;68:195-197
24. .Primär- und Sekundärprävention der zerebralen Ischämie. In Leitlinien für Diagnostik und Therapie in der Neurologie. Herausgegeben von der Kommission "Leitlinien der Deutschen Gesellschaft für Neurologie". Thieme Verlag Stuttgart:2008
25. Char D, Cuadra S, Ricotta J, Bilfinger T, Giron F, McLarty A, Krukenkamp I, Saltman A, Seifert F. Combined coronary artery bypass and carotid endarterectomy: Long-term results. *Cardiovasc Surg*. 2002;10:111-115
26. Ringleb PA, Allenberg J, Bruckmann H, Eckstein HH, Fraedrich G, Hartmann M, Hennerici M, Jansen O, Klein G, Kunze A, Marx P, Niederkorn K, Schmiedt W, Solymosi L, Stingele R, Zeumer H, Hacke W. 30 day results from the space trial of stent-protected angioplasty versus carotid endarterectomy in symptomatic patients: A randomised non-inferiority trial. *Lancet*. 2006;368:1239-1247
27. Hobson RW, Weiss DG, Fields WS, Goldstone J, Moore WS, Towne JB, Wright CB, and the Veterans Affairs Cooperative Study Group. Efficacy of carotid endarterectomy for asymptomatic carotid stenosis. *N Engl J Med*. 1993;328:221-227
28. European Carotid Surgery Trialists' Collaborative Group. Randomised trial of endarterectomy for recently symptomatic carotid stenosis: Final results of the mrc european carotid surgery trial (ecst). *Lancet*. 1998;351:1379-1387
29. Nicolaides AN, Kakkos SK, Griffin M, Sabetai M, Dhanjil S, Tegos T, Thomas DJ, Giannoukas A, Geroulakos G, Georgiou N, Francis S, Ioannidou E, Dore CJ. Severity of asymptomatic carotid stenosis and risk of ipsilateral hemispheric ischaemic events: Results from the acsrs study. *Eur J Vasc Endovasc Surg*. 2005;30:275-284

30. Thygesen K, Alpert JS, White HD. Universal definition of myocardial infarction. *Eur Heart J*. 2007;28:2525-2538

31. Molenberghs G, Verbeke G. Models for discrete longitudinal data. *Springer, Berlin*. 2005

# CABACS

## Coronary Artery Bypass graft surgery

## in patients with

## Asymptomatic Carotid Stenosis

## A Randomized Controlled Clinical Trial

**1. AMENDMENT** TO TRIAL PROTOCOL FROM 29.09.2010 VERSION 3.1

DATE OF THE AMENDMENT: 16.05.2012

EFFECTIVE DATE OF THE POLICY ENDORSEMENT

AFTER APPROVAL BY THE ETHICS COMMITTEE (EC) IN ESSEN AND APPROVAL IN EACH TRIAL SITE

**Coordinating Investigator (LKP):**
Prof. Dr. med. Christian Weimar
Department of Neurology; University of Duisburg-Essen
Hufelandstr. 55; 45147 Essen
Phone: 0201 723 2495; Fax: 0201 723 5919
e-mail: christian.weimar@uk-essen.de

**TRIAL COORDINATOR**
Dr. med. Stephan Knipp
Dept. of Thoracic and Cardiovascular Surgery; University of Duisburg-Essen
Hufelandstr. 55; 45147 Essen
Phone: 0201 723 4915; Fax: 0201 723 5451
e-mail: stephan.knipp@uk-essen.de

**AMENDMENT IN DETAIL**

All Amendments are marked as underline or crossedout

| Page | Former Description in the trial protocol | Amendment | explanatory statement |
|---|---|---|---|
| *3/17* | - | Male or female patients<br>Age ≥18 years | Until now the fact that only adult patients can be included in the trial has been described in the inclusion criteria “full legal capacity” |
| *3/17* | Myocardial infarction (NSTEMI or STEMI) within the past 7 days or hemodynamically unstable patients | NSTEMI within the past 48 hours, STEMI within the past 7 days or hemodynamically unstable patients | Non-ST Segment Elevation Myocardial Infarction-Acute Coronary Syndrome (NSTE-ACS) is the most frequent manifestation of ACS. Considering the large number of patients and the heterogeneity of NSTE-ACS, early risk stratification is important to identify patients at high immediate and long-term risk of death and cardiovascular events, in whom an early invasive strategy with its adjunctive medical therapy may reduce that risk. A substantial benefit with an early invasive strategy has only been proven in patients at high risk. Troponin elevation and ST depression at baseline appear to be among the most powerful individual predictors of benefit from invasive treatment (Mehta et al., JAMA 2005;292:2908-2917). In lower risk subsets of NSTE-ACS patients, angiography and subsequent revascularization can be delayed without increased risk but should be performed during the same hospital stay, preferably within 72 hours of admission. An invasive strategy always starts with angiography. After defining the anatomy and its associated risk features, a decision about the type of intervention can be made. No prospective RCT has specifically addressed the selection of mode of intervention in patients with NSTE-ACS. In stabilized patients after an episode of ACS, there is no reason to interpret differently the results from RCTs comparing the two revascularization |

| | | | |
|---|---|---|---|
| | | | methods in stable coronary artery disease (CAD). The mode of revascularization should be based on the severity and distribution of the CAD.<br>In the setting of STEMI, primary PCI is the treatment of choice for reperfusion. In cases of unfavourable anatomy for PCI or PCI failure, emergency CABG in evolving STEMI should only be considered when a very large area of myocardium is in jeopardy and surgical revascularization can be completed before this area becomes necrotic (i.e. in the initial 3-4 h). Current evidence points to an inverse relationship between surgical mortality and time elapsed since STEMI. When possible, in the absence of persistent pain or hemodynamic deterioration, a waiting period of 3-7 days appears to be the best compromise (Weiss et al., J Thorac Cardiovasc Surg 2008; 135: 503 -511). |
| *3/17* | Known high risk for cardiogenic embolism requiring anticoagulation (mechanical heart valve, chronic atrial fibrillation, left ventricular thrombus, left ventricular aneurysm) | ~~Known high risk for cardiogenic embolism requiring anticoagulation (mechanical heart valve, chronic atrial fibrillation, left ventricular thrombus, left ventricular aneurysm)~~ | The incidence of preoperative persistent/paroxysmal atrial fibrillation (AF) is reported to be as high as 20% in patients undergoing CABG surgery (Gammie et al., Ann Thorac Surg 2008;85:909-14). Studies have identified multiple risk factors for stroke after cardiac surgery (Hogue et al., Circulation 1999;100:642-647; Tarakji et al., JAMA 2011;375:381-390). With respect to the time when the patients develop strokes, new strokes after cardiac surgery may be classified as having occurred intraoperatively or postoperatively. In a cohort of 2972 cardiac surgical patients, prior neurological event, aortic atherosclerosis, and duration of cardiopulmonary bypass were independently associated with early stroke, whereas predictors of delayed stroke were prior neurological events, diabetes and aortic atherosclerosis. Atrial fibrillation had no impact on postoperative stroke rate unless it was accompanied by low cardiac output |

| | | | |
|---|---|---|---|
| | | | syndrome (Hogue et al., 1999). In a recent huge prospective study conducted from 1982 through 2009 at a single US academic medical center among 45,432 consecutive patients undergoing isolated primary or reoperative CABG surgery, older age and variables representing atherosclerotic burden were found to be risk factors for early and late stroke. Preoperative atrial fibrillation was a strong predictor of early (p<0.002, OR 2.4 [95% CI 1.38-4.2) and late (p<0.001, OR 3.0 [1.64-5.4]) postoperative stroke, while new-onset postoperative AF was less predictive (p<0.04, HR 0.57 [0.33-0.97]) (Tarakji et al., 2011). Notwithstanding the putative increase of perioperative stroke risk in CABG patients with AF, patients enrolled in the CABACS trial are randomly assigned to undergo CABG with or without CEA and the additional stroke risk related to concomittant AF will be equally distributed between both treatment groups. |
| *20* | The randomization list will be kept in safe and confidential custody at the ZKSE. | ~~The randomization list will be kept in safe and confidential custody at the ZKSE.~~ <u>A web based central randomization was chosen.</u> | Due to the web based central randomization the randomization list is not necessary |
| *29* | According to the Declaration of Helsinki every investigator should report all SAE to his Ethics Committee. In this trial with up to 40 sites, this reporting is counterproductive. A central reporting will improve the | According to the Declaration of Helsinki every investigator should report all SAE to his Ethics Committee. In this trial with up to 40 sites, this reporting is counterproductive. A central reporting | The patient recruitment is poorer than expected. As of May 11, 2012, 33 patients have been enrolled in the trial. Therefore a quarterly report of SAE to the Ethics Committee is not absolutely necessary. SAE resulting in death should continue to be reported separately. |

| | | | |
|---|---|---|---|
| | safety of all trial patients. With the Ethics Committee in Essen the following agreement was concluded:<br>A report (analogous to the annual safety report) will be sent to the independent Ethics Committee (University of Duisburg-Essen) and the sites. This report includes all SAE and OE. This report will be sent every 3 months (4 times a year).<br><br>SAEs resulting in death are to be reported separately to the Ethics Committee (University of Duisburg-Essen) within 14 days. | will improve the safety of all trial patients. With the Ethics Committee in Essen the following agreement was concluded:<br>A report (analogous to the annual safety report) will be sent to the independent Ethics Committee (University of Duisburg-Essen) and the sites. This report includes all SAE and OE. This report will be sent <u>every year or after inclusion of 100 patients.</u><br><br>SAEs resulting in death are to be reported separately to the Ethics Committee (University of Duisburg-Essen) within 14 days. | |

# CABACS

## Coronary Artery Bypass graft surgery

## in patients with

## Asymptomatic Carotid Stenosis

## A Randomized Controlled Clinical Trial

**2. AMENDMENT** TO TRIAL PROTOCOL FROM 29.09.2010 VERSION 3.1

DATE OF THE AMENDMENT: 04.07.2014

EFFECTIVE DATE OF THE POLICY ENDORSEMENT

AFTER APPROVAL BY THE ETHICS COMMITTEE (EC) IN ESSEN AND APPROVAL IN EACH TRIAL SITE

**Coordinating Investigator (LKP):**
Prof. Dr. med. Christian Weimar
Department of Neurology; University of Duisburg-Essen
Hufelandstr. 55; 45147 Essen
Phone: 0201 723 6503; Fax: 0201 723 6948
e-mail: christian.weimar@uk-essen.de

**TRIAL COORDINATOR**
Dr. med. Stephan Knipp
Dept. of Thoracic and Cardiovascular Surgery; University of Duisburg-Essen
Hufelandstr. 55; 45147 Essen
Phone: 0201 723 4915; Fax: 0201 723 5451
e-mail: stephan.knipp@uk-essen.de

**AMENDMENT IN DETAIL**

All Amendments are marked as underlined or ~~crossedout~~

| Page | Former Description in the trial protocol | Amendment | explanatory statement |
|---|---|---|---|
| 1/2 | Prof. Dr. med. Christian Weimar Department of Neurology; University of Duisburg-Essen Hufelandstr. 55; 45147 Essen Phone: 0201 723 2495; Fax: 0201 723 5919 e-mail: christian.weimar@uk-essen.de | Prof. Dr. med. Christian Weimar Department of Neurology; University of Duisburg-Essen Hufelandstr. 55; 45147 Essen Phone: 0201 723 6503; Fax: 0201 723 6948 e-mail: christian.weimar@uk-essen.de | The phone number of Prof. Weimar has changed and has to be modified in his contact data |
| *3/17* | - | Male or female patients<br>Age ≥18 years | Until now the fact that only adult patients can be included in the trial has been described in the inclusion criteria “full legal capacity” |
| *3/17* | Myocardial infarction (NSTEMI or STEMI) within the past 7 days or hemodynamically unstable patients | NSTEMI within the past 48 hours, STEMI within the past 7 days or hemodynamically unstable patients | Non-ST Segment Elevation Myocardial Infarction-Acute Coronary Syndrome (NSTE-ACS) is the most frequent manifestation of ACS. Considering the large number of patients and the heterogeneity of NSTE-ACS, early risk stratification is important to identify patients at high immediate and long-term risk of death and cardiovascular events, in whom an early invasive strategy with its adjunctive medical therapy may reduce that risk. A substantial benefit with an early invasive strategy has only been proven in patients at high risk. Troponin elevation and ST depression at baseline appear to be among the most powerful individual predictors of benefit from invasive treatment (Mehta et al., JAMA 2005;292:2908-2917). In lower risk subsets of NSTE-ACS patients, angiography and subsequent revascularization can be delayed without |

| | | | |
|---|---|---|---|
| | | | increased risk but should be performed during the same hospital stay, preferably within 72 hours of admission. An invasive strategy always starts with angiography. After defining the anatomy and its associated risk features, a decision about the type of intervention can be made. No prospective RCT has specifically addressed the selection of mode of intervention in patients with NSTE-ACS. In stabilized patients after an episode of ACS, there is no reason to interpret differently the results from RCTs comparing the two revascularization methods in stable coronary artery disease (CAD). The mode of revascularization should be based on the severity and distribution of the CAD.<br>In the setting of STEMI, primary PCI is the treatment of choice for reperfusion. In cases of unfavourable anatomy for PCI or PCI failure, emergency CABG in evolving STEMI should only be considered when a very large area of myocardium is in jeopardy and surgical revascularization can be completed before this area becomes necrotic (i.e. in the initial 3-4 h). Current evidence points to an inverse relationship between surgical mortality and time elapsed since STEMI. When possible, in the absence of persistent pain or hemodynamic deterioration, a waiting period of 3-7 days appears to be the best compromise (Weiss et al., J Thorac Cardiovasc Surg 2008; 135: 503 -511). |
| *3/17* | Known high risk for cardiogenic embolism requiring anticoagulation (mechanical heart valve, chronic atrial fibrillation, left ventricular thrombus, left ventricular aneurysm) | ~~Known high risk for cardiogenic embolism requiring anticoagulation (mechanical heart valve, chronic atrial fibrillation, left ventricular thrombus, left ventricular~~ | The incidence of preoperative persistent/paroxysmal atrial fibrillation (AF) is reported to be as high as 20% in patients undergoing CABG surgery (Gammie et al., Ann Thorac Surg 2008;85:909-14). Studies have identified multiple risk factors for stroke after cardiac surgery (Hogue et al., Circulation 1999;100:642-647; Tarakji et al., JAMA 2011;375:381-390). With respect to the time when the patients develop strokes, |

| | | | |
|---|---|---|---|
| | | ~~aneurysm)~~ | new strokes after cardiac surgery may be classified as having occurred intraoperatively or postoperatively. In a cohort of 2972 cardiac surgical patients, prior neurological event, aortic atherosclerosis, and duration of cardiopulmonary bypass were independently associated with early stroke, whereas predictors of delayed stroke were prior neurological events, diabetes and aortic atherosclerosis. Atrial fibrillation had no impact on postoperative stroke rate unless it was accompanied by low cardiac output syndrome (Hogue et al., 1999). In a recent huge prospective study conducted from 1982 through 2009 at a single US academic medical center among 45,432 consecutive patients undergoing isolated primary or reoperative CABG surgery, older age and variables representing atherosclerotic burden were found to be risk factors for early and late stroke. Preoperative atrial fibrillation was a strong predictor of early (p<0.002, OR 2.4 [95% CI 1.38-4.2) and late (p<0.001, OR 3.0 [1.64-5.4]) postoperative stroke, while new-onset postoperative AF was less predictive (p<0.04, HR 0.57 [0.33-0.97]) (Tarakji et al., 2011). Notwithstanding the putative increase of perioperative stroke risk in CABG patients with AF, patients enrolled in the CABACS trial are randomly assigned to undergo CABG with or without CEA and the additional stroke risk related to concomittant AF will be equally distributed between both treatment groups. |
| *20* | The randomization list will be kept in safe and confidential custody at the ZKSE. | ~~The randomization list will be kept in safe and confidential custody at the ZKSE.~~ A web based central randomization | Due to the web based central randomization the randomization list is not necessary |

| | | | |
|---|---|---|---|
| | | <u>was chosen.</u> | |
| *29* | According to the Declaration of Helsinki every investigator should report all SAE to his Ethics Committee. In this trial with up to 40 sites, this reporting is counterproductive. A central reporting will improve the safety of all trial patients. With the Ethics Committee in Essen the following agreement was concluded:<br>A report (analogous to the annual safety report) will be sent to the independent Ethics Committee (University of Duisburg-Essen) and the sites. This report includes all SAE and OE. This report will be sent every 3 months (4 times a year).<br><br>SAEs resulting in death are to be reported separately to the Ethics Committee (University of | According to the Declaration of Helsinki every investigator should report all SAE to his Ethics Committee. In this trial with up to 40 sites, this reporting is counterproductive. A central reporting will improve the safety of all trial patients. With the Ethics Committee in Essen the following agreement was concluded:<br>A report (analogous to the annual safety report) will be sent to the independent Ethics Committee (University of Duisburg-Essen) and the sites. This report includes all SAE and OE. This report will be sent <u>every year or after inclusion of 100 patients.</u><br><br>SAEs resulting in death are to be reported separately to the Ethics Committee | The patient recruitment is poorer than expected. As of May 11, 2012, 33 patients have been enrolled in the trial. Therefore a quarterly report of SAE to the Ethics Committee is not absolutely necessary. SAE resulting in death should continue to be reported separately. |

| | | | |
|---|---|---|---|
| | Duisburg-Essen) within 14 days. | (University of Duisburg-Essen) within 14 days. | |
