# Supplementary figures and images for: Safety of Simultaneous Coronary Artery Bypass Grafting and Carotid Endarterectomy Versus Isolated Coronary Artery Bypass Grafting: A Randomized Clinical Trial
Source: Stroke. 2017 Sep 15;48(10):2769–75. doi: 10.1161/STROKEAHA.117.017570 (PMC5610560; doi:10.1161/STROKEAHA.117.017570)

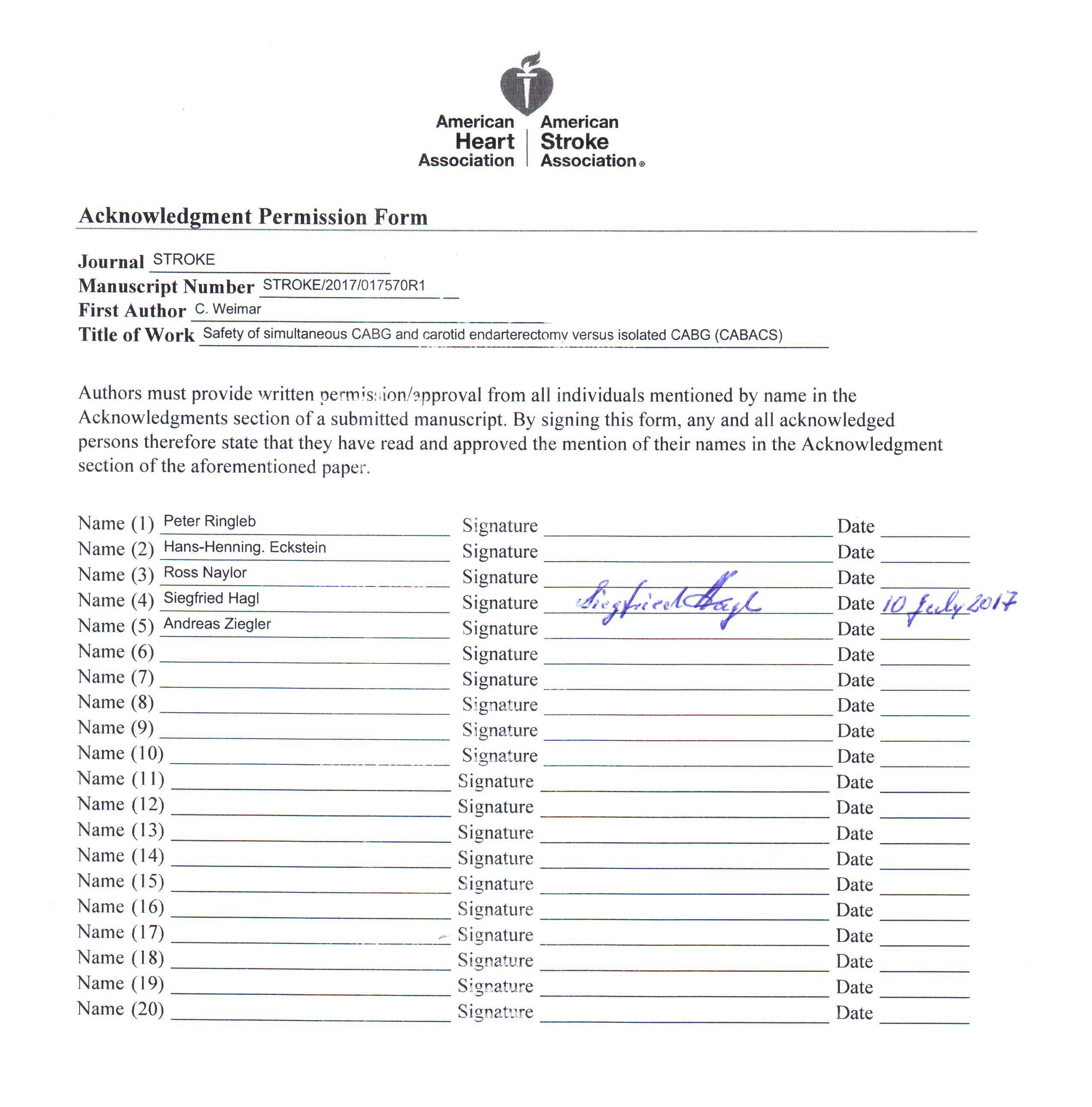

Supplement: Supplementary file 2 [file str-48-2769-s002.jpg]
