# Supplementary material for: Safety of Simultaneous Coronary Artery Bypass Grafting and Carotid Endarterectomy Versus Isolated Coronary Artery Bypass Grafting: A Randomized Clinical Trial
Source: Stroke. 2017 Sep 15;48(10):2769–75. doi: 10.1161/STROKEAHA.117.017570 (PMC5610560; doi:10.1161/STROKEAHA.117.017570)
Supplement: Supplementary file 3 [file str-48-2769-s003.pdf]

American Heart Association | American Stroke Association®

Acknowledgment Permission Form

Journal STROKE
Manuscript Number STROKE/2017/017570R1
First Author C. Weimar
Title of Work Safety of simultaneous CABG and carotid endarterectomy versus isolated CABG (CABACS)

Authors must provide written permission/approval from all individuals mentioned by name in the Acknowledgments section of a submitted manuscript. By signing this form, any and all acknowledged persons therefore state that they have read and approved the mention of their names in the Acknowledgment section of the aforementioned paper.

Name (1)	Peter Ringleb	Signature	Date
Name (2)	Hans-Henning. Eckstein	Signature	Date
Name (3)	Ross Naylor	Signature [illegible]	Date 10-7-17
Name (4)	Siegfried Hagl	Signature	Date
Name (5)	Andreas Ziegler	Signature	Date
Name (6)		Signature	Date
Name (7)		Signature	Date
Name (8)		Signature	Date
Name (9)		Signature	Date
Name (10)		Signature	Date
Name (11)		Signature	Date
Name (12)		Signature	Date
Name (13)		Signature	Date
Name (14)		Signature	Date
Name (15)		Signature	Date
Name (16)		Signature	Date
Name (17)		Signature	Date
Name (18)		Signature	Date
Name (19)		Signature	Date
Name (20)		Signature	Date
